# ANSID: A Solid-Phase Proteomic Approach for Identification and Relative Quantification of Aromatic Nitration Sites

**DOI:** 10.3389/fchem.2015.00070

**Published:** 2016-01-07

**Authors:** Tal Nuriel, Julia Whitehouse, Yuliang Ma, Emily J. Mercer, Neil Brown, Steven S. Gross

**Affiliations:** ^1^Department of Pharmacology, Weill Cornell Medical CollegeNew York, NY, USA; ^2^Department of Pathology and Cell Biology, Taub Institute for Research on Alzheimer's Disease and the Aging Brain, Columbia University Medical CollegeNew York, NY, USA; ^3^Department of Surgery, Weill Cornell Medical CollegeNew York, NY, USA

**Keywords:** nitrotyrosine, nitrotryptophan, nitric oxide, proteomics, post-translational modifications, peroxynitrite, nitrosative stress

## Abstract

Nitration of tyrosine and other aromatic amino acid residues in proteins occurs in the setting of inflammatory, neurodegenerative, and cardiovascular diseases—importantly, this modification has been implicated in the pathogenesis of diverse diseases and the physiological process of aging. To understand the biological consequences of aromatic nitration in both health and disease, it is critical to molecularly identify the proteins that undergo nitration, specify their cognate modification sites and quantify their extent of nitration. To date, unbiased identification of nitrated proteins has often involved painstaking 2D-gel electrophoresis followed by Western Blotting with an anti-nitrotyrosine antibody for detection. Apart from being relatively slow and laborious, this method suffers from limited coverage, the potential for false-positive identifications, and failure to reveal specific amino acid modification sites. To overcome these shortcomings, we have developed a solid-phase, chemical-capture approach for unbiased and high-throughput discovery of nitrotyrosine and nitrotryptophan sites in proteins. Utilizing this method, we have successfully identified several endogenously nitrated proteins in rat brain and a total of 244 nitrated peptides from 145 proteins following *in vitro* exposure of rat brain homogenates to the nitrating agent peroxynitrite (1 mM). As expected, Tyr residues constituted the great majority of peroxynitrite-mediated protein nitration sites; however, we were surprised to discover several brain proteins that contain nitrated Trp residues. By incorporating a stable-isotope labeling step, this new Aromatic Nitration Site IDentification (ANSID) method was also adapted for relative quantification of nitration site abundances in proteins. Application of the ANSID method offers great potential to advance our understanding of the role of protein nitration in disease pathogenesis and normal physiology.

## Introduction

Aromatic nitration is a posttranslational modification that involves the addition of a nitro (NO_2_) group to the benzene ring of an aromatic amino acid residue in proteins (Alvarez and Radi, 2003). This modification most commonly occurs during pathological conditions where high levels of reactive nitrogen species (RNS) are formed, primarily peroxynitrite (ONOO^−^), arising from the near diffusion-limited reaction of nitric oxide (NO) with superoxide (${\text{O}}_{2}^{-}$) (Pacher et al., 2007). The most widely recognized protein aromatic nitration event occurs on Tyr residues, yielding proteinaceous 3-nitrotyrosine (3-NT), which often accumulates in the setting of neurodegenerative (Ferrante et al., 1999; Castegna et al., 2003; Sacksteder et al., 2006; Sultana et al., 2006; Reynolds et al., 2007) and cardiovascular diseases (Patel et al., 2000; Rubbo et al., 2000; Turko and Murad, 2002; Harrison et al., 2003; Peluffo and Radi, 2007), as well as during the physiological process of aging (van der Loo et al., 2000; Beal, 2002; Kanski et al., 2005b). This modification has the dual chemical consequences of both increasing the Tyr residue's steric bulk and decreasing the pKa of its hydroxyl group (from about 10.1 to 7.2)—potentially resulting in altered interactions with protein binding partners and/or perturbed activity of an enzyme (Abello et al., 2009). In comparison with nitration of Tyr, much less is known about the prevalence and potential effects of Trp, His, and Phe nitration in proteins. Notwithstanding, there is evidence that all of these modifications can occur under pathological conditions (Ferger et al., 2001; Ishii et al., 2007; Rebrin et al., 2007) and result in altered protein activities (Alvarez et al., 2004; Yamakura et al., 2005; Rebrin et al., 2007).

In order to elucidate the contribution that aromatic amino acid nitration plays in disease pathogenesis, it is important to recognize the relevant proteins that undergo nitration in a given pathological condition, as well as their specific sites of nitration. To date, most attempts to identify nitrated proteins in an unbiased manner have utilized 2D-gel electrophoresis using an anti-3-NT antibody (Castegna et al., 2003; Kanski et al., 2005a; Sultana et al., 2006). However, this approach possesses numerous shortcomings, including limited coverage, the potential for false-positive identifications and failure to identify the specific sites of nitration (Kanski and Schöneich, 2005). Furthermore, since the detection of nitrated proteins is performed using a 3-NT-specific antibody, this approach is unable to identify proteins that are nitrated on aromatic amino acid residues other than Tyr.

In attempt to overcome these limitations, several groups have sought to develop an in-solution enrichment strategy for LC-MS/MS based unbiased identification of nitrated proteins and their sites of nitration, similar to the SNOSID method that was developed in our laboratory for the unbiased discovery of S-nitrosylated proteins and specification of their cognate sites of Cys modification (Hao et al., 2006). These earlier attempts at establishing proteomic strategies for unbiased discovery of protein nitration sites, including some reported during the development of the present ANSID method, have demonstrated some success in identifying nitrated proteins. However, these are limited to the identification of synthesized pure nitrated proteins or peptides (Tsumoto et al., 2010), pure nitrated proteins or peptides that were spiked into complex protein mixtures (Nikov et al., 2003; Abello et al., 2010; Dremina et al., 2011; Guo et al., 2012), and relatively high concentrations of nitrated proteins or peptides that were created by ONOO^−^-treated human plasma (Prokai-Tatrai et al., 2011) or rat brain homogenates (Zhang et al., 2007). In addition, one method (Robinson and Evans, 2012) has accomplished the feat of detecting low-level endogenous protein nitration (in mouse spleen homogenates). However, it should be noted that this method utilized an in-house, chemically synthesized tagging reagent that would limit access to this technology.

In the current report, we describe an optimized approach with increased sensitivity and selectivity, and which utilizes inexpensive commercially available reagents, for the unbiased identification of nitrated proteins and their sites of nitration. We term this new approach the ANSID method, *for* Aromatic Nitration Site IDentification. Using the ANSID method, we could successfully identify endogenously nitrated rat brain protein, as well as >100 proteins that are most prone to peroxynitrite-mediated nitration, along with their cognate modification sites. Notably, these included several Trp-nitrated residues in addition to the more commonly observed Tyr-nitrated residues. As a further advance, we have used stable isotope labeling to adapt the ANSID method for relative quantification of nitration site abundance in proteins. The ANSID method offers a significant advancement over the previously reported approaches due to its ease of use, ability to specifically and sensitively recognize nitrated proteins, identify their sites of nitration, and ascertain how levels and patterns of protein nitration may change in disparate physiological and pathophysiological settings.

## Materials and methods

### Reagents

All chemicals and reagents were purchased from Sigma-Aldrich (St. Louis, MO) in the best available grade, unless otherwise noted. Rat brains, stripped of the meninges, were obtained from Pel-Freez Biologicals (Rogers, AR). Sodium peroxynitrite (100–200 mM in 4.7% sodium hydroxide) was obtained from EMD Millipore (Billerica, MA) and was aliquoted and stored at −80°C for up to 6 months and then thawed immediately before use. Sulfo-NHS-SS-Biotin and Aminolink aldehyde-agarose beads were purchased from Thermo Fisher Scientific (Waltham, MA), and Strata-X C18 columns were purchased from Phenomenex (Torrance, CA). The histone H1.2-derived peptide, ALAAAG(Y-NO_2_)DVEK, was custom-synthesized by Genscript (Piscataway, NJ).

### Sample preparation for ANSID analysis

For nitrated brain homogenate samples, 10 rat brains were washed 3-times with 1 × PBS and placed in 15 ml ice-cold lysis buffer (100 mM sodium phosphate, pH 7.7, 150 mM NaCl, 0.4% Triton X-100, 1 mM protease inhibitor cocktail). Homogenization was performed on ice using a Tissue-Terror (Biospec, Bartlesville, OK) at half-maximal speed for 3 intervals of 30 s each. The homogenate was aliquoted into Eppendorf tubes and centrifuged (13,400 × *g*) for 15 min at 4°C and supernatants were collected. Total protein concentration was quantified with the DC protein assay (Bio-Rad), and the pooled supernatant was distributed to tubes, each containing 40 mg of protein.

In experiments where exogenous protein nitration was performed, samples were diluted with lysis buffer to 8.75 ml (4.57 mg protein/ml), and nitration was elicited by adding freshly-thawed ONOO^−^ at the desired concentration and incubation for 30 min at room temperature with gentle rocking. Following nitration, 1.5 ml of 20% sodium dodecyl sulfate (SDS) was added to each sample (4 mg/ml protein, 2.5% SDS), and proteins were denatured by boiling in a water bath for 5 min at 95°C. Cys residues were then reduced with 20 mM dithiothreitol (DTT) for 1 h at 60°C and alkylated with 80 mM iodoacetamide for 45 min at room temperature in the dark. To remove excess reagents, proteins were precipitated with 2-volumes of ice-cold acetone and pelleted by centrifugation at 2600 × *g* for 5 min. Protein pellets were resuspended in 7.5 ml of 100 mM sodium phosphate buffer, pH 7.7 to bring the total volume up to 8 ml (5 mg protein/ml). To digest proteins, TPCK-modified trypsin was added at a ratio of 50:1 protein:trypsin (wt:wt), and the samples were rotated overnight at room temperature.

### Amine-blocking of Lys- and N-terminal residues and reduction of nitro groups for ANSID analysis

Peptide extracts were aliquoted into 5 mg quantities (1 ml each) in 15 ml tubes. Dimethylation of Lys- and N-terminal amino groups was performed by adding 100 mM formaldehyde, 200 mM dimethylamine borane (DMAB), and 100 mM hydrochloric acid (HCl) (to maintain the pH at 7–8), with gentle mixing after the addition of each reagent, followed by incubation for 10 min at 60°C with vigorous shaking. A second round of dimethylation was then performed to ensure complete blocking of primary amines. Because formaldehyde and DMAB are highly toxic, dimethylation, as well as the following two steps, were performed under the hood. Following dimethylation, the formaldehyde/DMAB reaction was quenched by the addition of 200 mM glycine and incubation for 10 min at 60°C with vigorous shaking. Nitro-groups present on aromatic amino acid residues were reduced to amino groups by incubating with 30 mM DTT and 250 μM hemin-agarose beads for 10 min in a 95°C water bath. Following reduction, hemin-agarose beads were removed by centrifugation at 2600 × *g* for 2 min, and the remaining reagents were removed by solid phase extraction (SPE) with a 30 mg resin Strata-X C18 column. Purified peptides were eluted from the C18 resin with 80% acetonitrile (ACN) in 25 mM sodium bicarbonate, pH 8. Following C18 clean up (i.e., removal of formaldehyde and DMAB), samples were removed from the chemical hood and further steps were performed on the lab bench. Aminolink aldehyde-agarose beads were washed twice in ACN and then resuspended 1:1 in 80% ACN in 25 mM sodium bicarbonate, pH 8 for peptide capture. For this purpose, 500 μl of the 1:1 bead-slurry was added to each sample, and the beads were rotated for 1 h at room temperature. Beads were washed in the cold room (to prevent hydrolysis of the imine-bonded peptide beads) 5-times with ice-cold wash buffer #1 (25 mM sodium bicarbonate, 600 mM NaCl; pH 8.0) and then 5-times with ice-cold wash buffer #2 (25 mM sodium bicarbonate, 20% ACN; pH 8.0). Captured peptides were eluted by adding 500 μl of 0.1% trifluoroacetic acid (TFA) and rotation for 1 h at room temperature. Finally, the eluted peptides were vacuum-centrifuged in a SpeedVac at 60°C to near-dryness and diluted to a final volume of 30 μl with 0.1% TFA for analysis by nanoflow liquid chromatography-tandem mass spectrometry (nanoLC-MS/MS).

### ANSID optimization with a model nitrated peptide

A custom-made histone H1.2-derived peptide, ALAAAG(Y-NO_2_)DVEK, was solubilized in 100 mM sodium phosphate buffer (pH 7.7) at a concentration of 5 mg/ml and stored at −80°C in 100 μl aliquots for up to 6 months prior to use. For dimethylation and hemin/DTT-reduction of 3-NT residues, the reactions were carried out as described above. For acetylation, 100 μl of the solubilized peptide was treated two times with 100 mM acetic anhydride at pH 11, each time with a 1 h incubation at 37°C with vigorous shaking. For sodium dithionite reduction of 3-NT residues, 100 μl of the dimethylated peptide was treated with 5 mM sodium dithionite for 10 min at room temperature with gentle rocking. For analysis, the peptide solution was diluted 1:100 in 0.1% TFA and then detected by nanoLC-MS/MS.

### ANSID optimization with NO_2_-BSA spiked into untreated rat brain homogenate

Bovine serum albumin (BSA) was solubilized in 100 mM sodium phosphate buffer, pH 7.7, at a concentration of 4 mg/ml. For nitration, 10 ml of 4 mg/ml BSA was treated with 1 mM of freshly-thawed ONOO^−^ and the resulting samples were incubated for 30 min at room temperature with gentle rocking. To assess 3-NT peptide detection in a complex biological mixture, NO2-BSA was spiked into a 4 mg/ml untreated rat brain homogenate at a concentration of 100 μg NO2-BSA per mg total protein. For the aldehyde-agarose bead-mediated ANSID, 5 mg of total protein was used, and the method was performed as described above. For the Sulfo-NHS-SS-Biotin-mediated ANSID, 20 mg of total protein was used and the ANSID was performed with several modifications. The Cys reduction and alkylation was performed as described above, followed immediately by Lys- and N-terminal dimethylation and nitro-group reduction on the intact proteins. To remove the excess reagents from proteins, each protein sample was cleaned up using a Zeba spin desalting column as described by the vendor's protocol (Thermo Fisher Scientific). The proteins were then treated with 4 mM Sulfo-NHS-SS-Biotin for 1 h at 37°C with vigorous shaking. To reverse hydroxyl-group modifications, the samples were treated with 0.5 M hydroxylamine at pH 8.0 for 10 min at 37°C with vigorous shaking and were again run through a Zeba spin desalting column to remove the excess reagents. Proteins were digested overnight at room temperature with TPCK-modified trypsin at a ratio of 50:1 protein:trypsin (wt:wt), after which the reactions were stopped by addition of 500 μM phenylmethylsulfonyl floride (PMSF). Prior to peptide capture, streptavidin-sepharose beads (GE, Piscataway, NJ) were washed twice in 25 mM ammonium bicarbonate, pH 8.0, and then resuspended 1:1 in 25 mM ammonium bicarbonate, pH 8.0. For capture, 100 μl of the 1:1 bead-slurry was added to each sample, and the beads were rotated for 1 h at room temperature. Beads were washed five times at room temperature with wash buffer #1 (25 mM ammonium bicarbonate, pH 8, 600 mM NaCl) and five times with wash buffer #2 (25 mM ammonium bicarbonate, pH 8, 20% acetonitrile). Captured peptides were then eluted by adding 150 μl of 100 mM β-mercaptoethanol in 15 mM ammonium bicarbonate, pH 8, and rotating at room temperature for 30 min. The eluted peptides were vacuum-centrifuged to near-dryness, diluted to a final volume of 30 μl with 0.1% TFA and analyzed by nanoLC-MS/MS.

### Isotope-coded ANSID approach for relative quantification of nitrated peptides

Rat brain homogenates were treated with 1 mM ONOO^−^, as described above, and then a portion of this homogenate was diluted in untreated rat brain homogenates to give ratios of 1:1, 1:4, 1:9, or 1:19 (nitrated homogenate:non-nitrated homogenate). Samples were analyzed in quadruplicate: comparing four samples containing 5 mg undiluted nitrated rat brain homogenate with each and four samples containing 5 mg of 1:1, 1:4, 1:9, or 1:19 dilutions of nitrated rat brain homogenate in untreated rat brain homogenate. For each sample, initial steps in the ANSID was performed as described above. However, for the dimethylation step, the diluted samples were treated with 100 mM heavy isotope-labeled formaldehyde (C^13^D_2_O; Sigma), while the undiluted samples were treated with only light isotope-containing formaldehyde (CH_2_O). Each sample was then treated with 200 mM glycine to quench the dimethylation reaction. At this point, each of the undiluted samples were pooled with one of the four diluted samples. The rest of the ANSID method was then continued as described above. Peptide abundances were quantified using nanoLC-MS/MS, by measuring the area under each ion current peak using Mass Hunter Qualitative Analysis software version B.03.01 (Agilent).

### Peptide identification by nanoLC-MS/MS

nanoLC-MS/MS was used to identify both the modified histone H1.2-derived peptide and the ANSID-enriched peptides from NO2-BSA and ONOO^−^-treated or untreated rat brain homogenates. Peptides were analyzed using a 6520 accurate-mass quadrupole-time of flight (Q-TOF) mass spectrometer coupled to a chip cube with an on-chip C18 column (Agilent Technologies, Santa Clara, CA). The mobile phases were 0.1% formic acid in water (solvent A) and 0.1% formic acid in 90% acetonitrile (solvent B). Eight microliters of each sample was injected onto either a 4 mm 40 nl Zorbax 300SB-C18 or a 360 nl Polaris 300SB-C18 enrichment column at a flow rate of 4 μl/min, and peptides were resolved on either a 0.075 × 43 mm Zorbax 300SB-C18 or a 0.075 × 150 mm Polaris 300SB-C18 analytical column at a flow rate of 0.4 μl/min. Peptide elution utilized solvent gradients, as follows: 3–25% solvent B for 10 min for histone H1.2-derived peptides, 3–50% solvent B for 20 min for NO2-BSA peptides enriched using the Sulfo-NHS-SS-Biotin-mediated ANSID, 3–45% solvent B for 24 min for NO2-BSA peptides enriched using the aldehyde-agarose beads-mediated ANSID and 3–40% solvent B for 52 min for peptides from ONOO^−^-treated or untreated rat brain homogenates. Non-eluting peptides were then displaced by treatment with 90% solvent B for 2–5 min. Mass spectra were acquired in automated data-dependent acquisition mode at 0.5 s intervals, and MS/MS scans were performed on the four most intense ions for each MS scan. Peptides were identified using Spectrum Mill software version A.03.03 (Agilent). Peptide files were searched against a regularly updated Swiss-Prot database, with a peak intensity threshold of 50%, precursor mass tolerance of 20 ppm and a product mass tolerance of 50 ppm. For each sample, four separate searches were performed. In each search, a fixed modification was set for dimethylated N-terminal residues and variable modifications for carbamidomethylated Cys residues, oxidized Met residues and dimethylated Lys residues, plus an additional variable modification for either NH_2_-Tyr, NH_2_-Trp, NH_2_-His, or NH_2_-Phe residues. To assess the level of non-specific peptide capture, additional searches were performed using a reverse Swill-Prot database with a fixed mono-methylated N-terminal modification or with no modification present on the N-terminal. For all searches, peptides with a Spectrum Mill score of 9 or higher were considered valid, which provides a false positive discovery rate of < 2% (calculated using a forward/reverse Swiss-Prot database).

## Results

**Figure 1:**
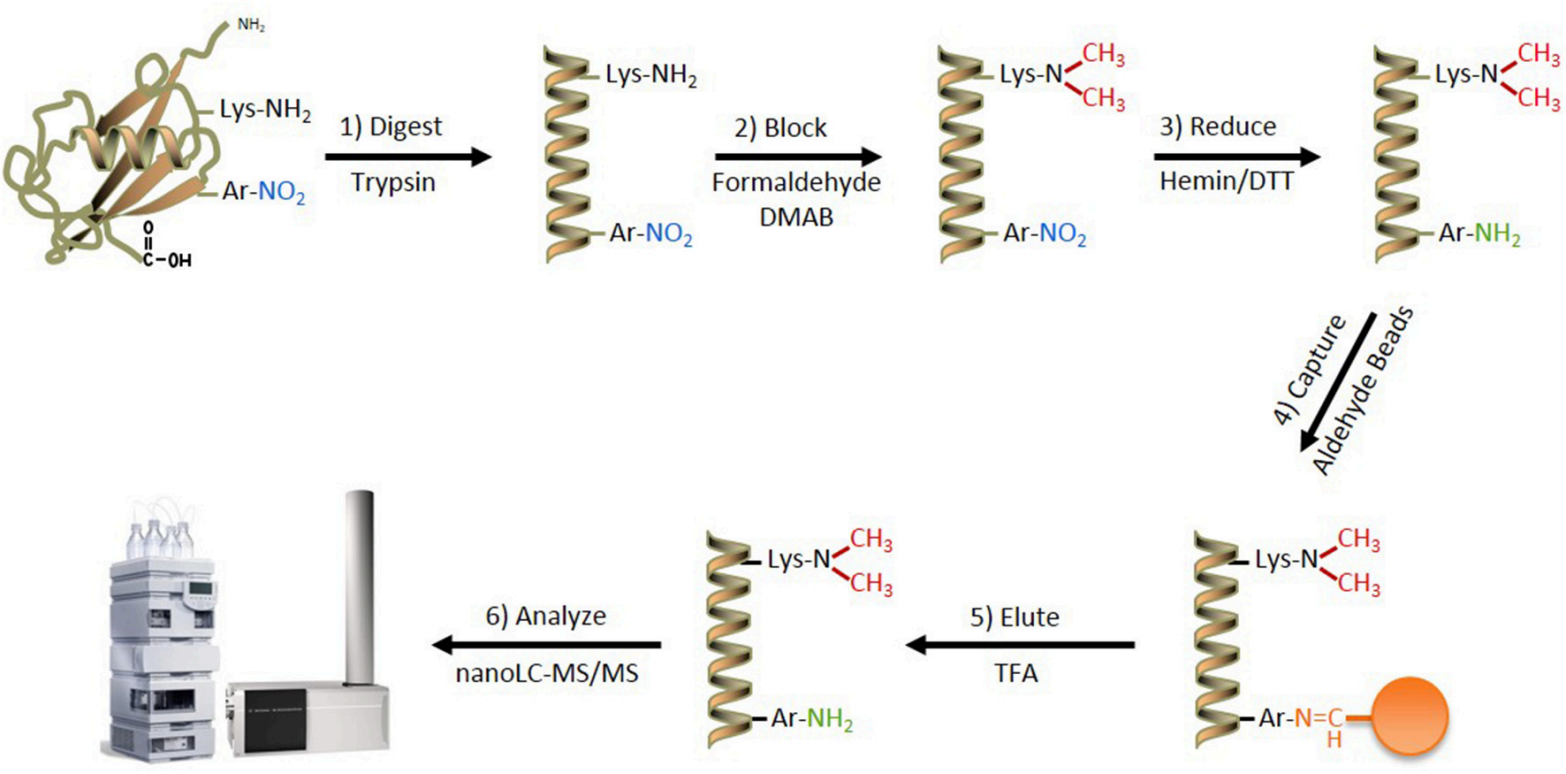
**The ANSID approach for aromatic nitration site identification**. A schematic of the ANSID method. Proteins are first digested with trypsin, followed by Lys- and N-terminal amine-blocking through reductive dimethylation with formaldehyde and dimethylamine borane. The nitro-aromatic residues are then reduced to produce amines with hemin-agarose beads in the presence of DTT, followed by capture and enrichment of the amino-aromatic amino acid-containing peptides using aldehyde-agarose beads. The captured peptides are then eluted with TFA and analyzed by nanoLC-MS/MS. Abbreviations: DMAB, dimethylamine borane; DTT, dithiothreitol; TFA, trifluoroacetic acid.

The most successful previous attempts at developing an in-solution enrichment strategy for unbiased identification of nitrated proteins and their cognate sites of nitration have relied on a stepwise procedure, comprising: (1) alkylation of protein or peptide amine moieties on Lys- and N-terminal amino acids, (2) chemical reduction of all aromatic ring nitro-groups to amino-groups, and (3) capture and enrichment strategies that takes advantage of the newly introduced amino group (Zhang et al., 2007; Abello et al., 2010; Tsumoto et al., 2010; Prokai-Tatrai et al., 2011; Guo et al., 2012; Robinson and Evans, 2012). The ANSID approach used in the present study followed this general strategy (Figure 1), however, our protocol was optimized and enhanced by the incorporation of novel strategies and reagents that provide increased specificity, sensitivity and relative quantification of protein nitration sites.

### Blocking of Lys- and N-terminal amines with formaldehyde and DMAB

**Figure 2:**
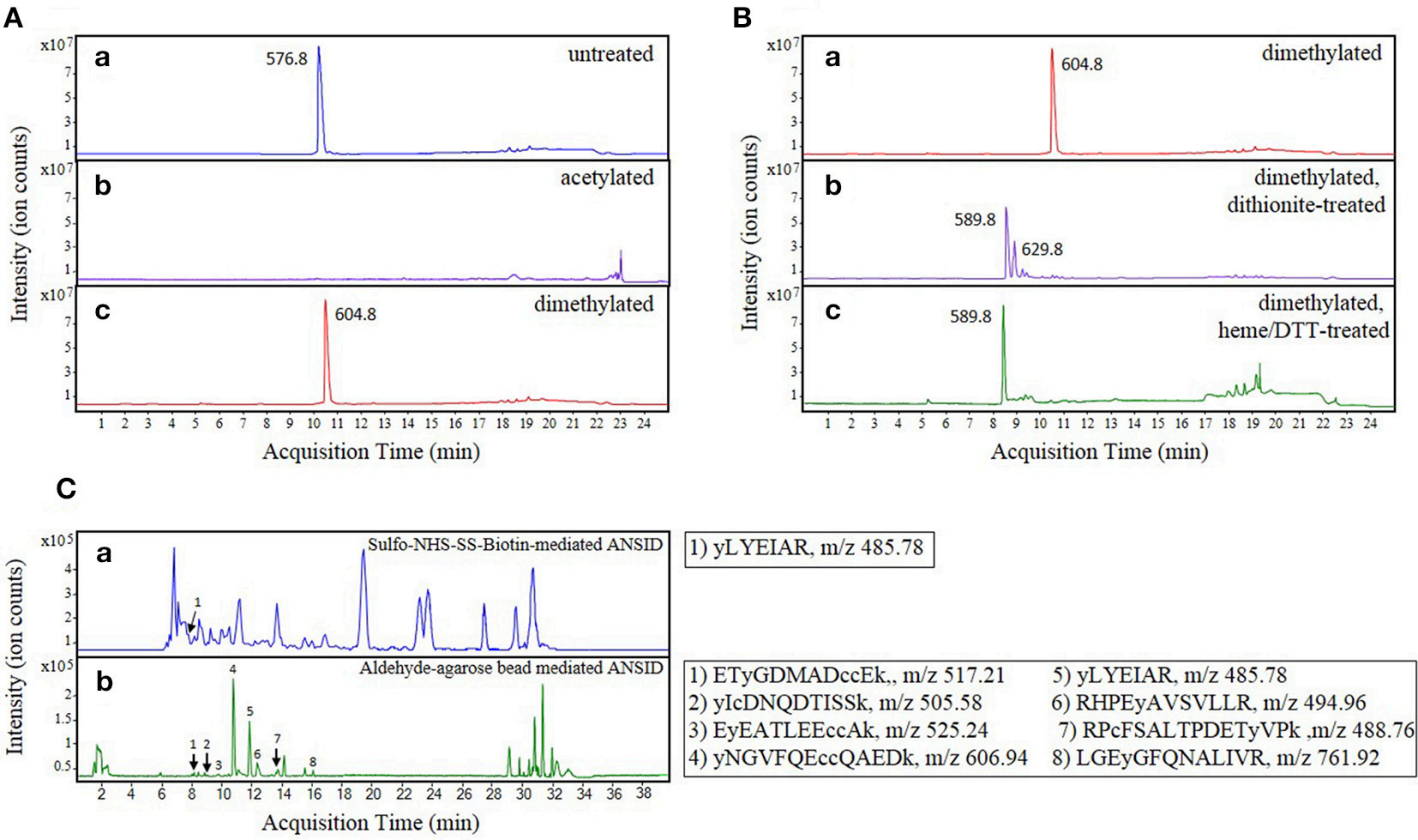
**Optimization of the ANSID protocol**. Base peak chromatograms (BPCs) showing the results of optimization experiments for **(A)** the amine-blocking step, **(B)** the nitro group reducing step, and **(C)** the amine-specific tagging/capture step. In Panel **(A)**, a pure synthetic peptide, ALAAG(Y-NO_2_)DVEK (*spectrum a*), derived from histone H1.2, was modified by either acetylation with acetic anhydride (*spectrum b*) or reductive dimethylation with formaldehyde and dimethylamine borane (*spectrum c*). When the unmodified peptide (m/z 576.8) was modified by acetylation, it became undetectable by MS (presumably due to loss of the essential positive charge), whereas reductive dimethylation resulted in the near-complete modification of the peptide to its dimethylated form (m/z 604.8) and did not interfere with its discovery by MS. In Panel **(B)**, the nitrotyrosine group on the dimethylated histone H1.2-derived nitrated peptide (*spectrum a*) was reduced to aminotyrosine with either sodium dithionite (*spectrum b*) or hemin-agarose beads and dithiothreitol (*spectrum c*). While reduction with sodium dithionite caused the formation of a plus 80-dalton side product (m/z 629.8) in addition to the reduced dimethylated peptide (m/z 589.8), reduction with hemin-agarose beads and dithiothreitol resulted in near-complete reduction of nitrotyrosine without the significant formation of side-products. In Panel **(C)**, the ANSID was performed on nitrated BSA spiked into untreated rat brain homogenate at a concentration of 100 μg NO_2_BSA per mg total protein, using either amine-specific tagging with Sulfo-NHS-SS-biotin and then affinity capture with streptavidin-agarose beads (*spectrum a*) or direct amine-specific capture with aldehyde-agarose beads (*sectrum b*). While the Sulfo-NHS-SS-biotin-mediated ANSID, which was performed on 20 mg total protein, resulted in the identification of only one nitrated BSA peptide, the aldehyde-agarose-mediated ANSID, which was performed on only 1 mg of total protein, resulted in the identification of eight nitrated BSA peptides.

In order to block Lys- and N-terminal amines, previously reported methods have employed acetylation with either acetic anhydride or NHS-acetate (Zhang et al., 2007; Abello et al., 2010; Tsumoto et al., 2010). However, this strategy interferes with the detection of Lys-containing peptides since acetylation removes a positive charge that otherwise contributes importantly to peptide ionization and detection by MS in positive ion mode (Patterson, 2001). This is especially problematic for MS-based sequencing of tryptic peptides that are cleaved C-terminally at Lys residues, and thus devoid of other basic residues that can potentially carry a positive charge. To assess the severity of this predicted shortcoming, we investigated the effect of acetic anhydride mediated acetylation on the detection of a synthetic histone H1.2-derived peptide, in which the only resident basic amino acid is a C-terminal Lys (Figure 2A, compare spectra a and b). In accord with expectations, this peptide was no longer observed after Lys acetylation when using positive-ion LC-MS for detection.

To mitigate against this MS signal loss that arises from acetylation-induced neutralization of positive charge on amine residues of peptides, we instead utilized reductive dimethylation with formaldehyde and DMAB as an amine-blocking strategy. Notably, reductive dimethylation involves two sequential methyl-group additions, where each methylation is introduced via two sequential chemical steps: first, the amine group is converted to a reversible imine bond (N = CH_2_) by reaction with formaldehyde; second, the resulting imine is reduced to N-CH_3_ by reaction with DMAB (Gidley and Sanders, 1982; Rayment, 1997). Because reductive dimethylation retains the amine's positive charge under acidic pH (Means and Feeney, 1995), sensitive identification of the modified peptides by positive ion detection mode is preserved.

As shown in Figure 2A, when the above-mentioned histone H1.2-derived model peptide was incubated with formaldehyde and DMAB for 10 min at 60°C, we observed complete dimethylation (compare spectra a and c). Thus, in addition to allowing for retention of positive charges on Lys- and N-terminal protein and peptide residues, reductive dimethylation with formaldehyde and DMAB was confirmed to be efficient and rapid. Furthermore, facile quenching of any unreacted formaldehyde is achieved by incubation with excess glycine, thereby eliminating the need for a sample purification step and associated sample losses that may otherwise occur.

### Reduction of nitro-groups with hemin/DTT

Previously published strategies for unbiased identification of nitrated proteins and sites of nitration utilized sodium dithionite for reduction of 3-NT residues to 3-aminotyrosine (3-AT) (Nikov et al., 2003; Zhang et al., 2007; Abello et al., 2010; Tsumoto et al., 2010; Dremina et al., 2011; Prokai-Tatrai et al., 2011; Guo et al., 2012; Robinson and Evans, 2012). However, as we previously showed (Nuriel et al., 2008), in addition to reducing 3-NT to 3-AT in a synthetic histone H1.2-derived peptide, treatment with sodium dithionite also resulted in accumulation of a significant side-product (Figure 2B, compare spectra a to b), which we identified as a product of O-sulfation on the hydroxyl group of a newly-formed 3-AT residue, a reaction that has been previously described (Ghesquière et al., 2006). Upon further studies, we found that this sulfated and dimethylated peptide species is unavailable for reaction with amine-reactive reagents (data not shown) and accordingly, this side-reaction product precludes the identification by MS of a significant fraction of 3-AT-containing peptides.

To oppose this loss of sensitivity when using dithionite as reductant, we sought to establish an alternative approach for the reduction of aromatic nitro- to amino- groups. Previously, Balabanli et al. reported that, in the presence of thiol-containing reagents, relatively small amounts of heme-containing proteins and reagents are capable of reducing 3-NT to 3-AT when this reaction is performed at elevated temperature (Balabanli et al., 1999). Therefore, we evaluated this heme/thiol reaction for efficient nitro-reduction step in our protocol, again using the histone H1.2-derived peptide to monitor reaction progression. As shown in Figure 2B (compare spectra a to c), incubating the dimethylated histone H1.2-derived peptide with hemin-agarose beads and DTT at 95°C for 10 min resulted in the complete reduction of 3-NT to 3-AT, with no other MS-observed side-products. In addition to increased efficiency of reaction, a further advantage of this hemin/DTT reduction step is that hemin-agarose beads can be easily removed by centrifugation, and the peptide reaction products can be efficiently purified free of residual reagents by selective binding of peptides to a C18 spin-column.

### Capture of amino-aromatic amino acid-containing peptides with aldehyde-agarose beads

Previously published methods for unbiased identification of nitrated proteins and sites of nitration have utilized some form of N-hydroxysuccinimide (NHS)-containing reagent to either tag or directly capture the 3-AT-containing proteins or peptides that result after 3-NT reduction (Nikov et al., 2003; Zhang et al., 2007; Tsumoto et al., 2010; Prokai-Tatrai et al., 2011; Guo et al., 2012). However, in addition to reacting with primary amines, NHS reagents can also react non-specifically with the guanidinium groups of Arg residues (Miller et al., 1997) and with the hydroxyl groups of Ser, Thr, and Tyr residues that lie in close proximity to His residues (Miller, 1996; Miller et al., 1997; Kalkhof and Sinz, 2008).

To test the efficiency of NHS-containing reagents for use in an amine-specific enrichment step, we assessed the utility of this strategy for detection of 3-NT in 1 mM ONOO^−^-treated BSA spiked into untreated rat brain homogenate (at a concentration of 100 μg NO2-BSA per mg total protein). As described in Materials and Methods Section, we performed a modified ANSID approach on the NO_2_-BSA/rat brain homogenate mixture that included the affinity tagging of amino-aromatic groups with Sulfo-NHS-SS-biotin, followed by capture of the biotinylated peptides with streptavidin-sepharose beads. As shown in Figure 2C (spectrum a), using this Sulfo-NHS-SS-biotin-mediated ANSID approach we were only able to identify a single nitrated BSA peptide by MS, and this was only achieved with a large amount (20 mg) of total protein.

Due to the apparent inefficiency of this Sulfo-NHS-SS-biotin-mediated ANSID, we searched for an alternative amine-reactive reagent that could be used for more efficient enrichment. Since our initial amine-blocking step utilized reductive dimethylation with formaldehyde and DMAB, we sought to evaluate the potential of a similar aldehyde-driven mechanism for amine-specific enrichment. As described previously, aldehyde reagents can be used to create a stable covalent bond with primary amines through a two-step mechanism, wherein the aldehyde reagent first reacts with the amine to form a labile imine bond, and then a borane reagent is used to reduce that imine bond to an stable bond. However, because we require the captured peptides to be eluted for shotgun identification by MS, an irreversible covalent linkage would not serve for our enrichment step. Instead, we hypothesized that if we captured the amino-aromatic-containing peptides with aldehyde-agarose beads in the absence of a borane reagent (thus stopping the reaction after the formation of the labile imine bond), we could wash away non-specifically-bound peptides in the cold (i.e., at 4°C, to limit hydrolysis of the imine bond), followed by elution of the captured peptides at acidic pH. Notably, because these acid-eluted peptides would possess an amine moiety on their formerly nitrated aromatic amino acid residues, they can be distinguished from unmodified aromatic amino acids by LC-MS/MS.

As with the Sulfo-NHS-SS-biotin-mediated ANSID, we tested this aldehyde-agarose-mediated ANSID using 1 mM ONOO^−^-treated BSA spiked into untreated rat brain homogenate at a concentration of 100 μg NO2-BSA per mg total protein. As shown in Figure 2C (spectrum b), performing the ANSID using aldehyde-agarose beads resulted in markedly greater specificity and selectivity than had previously been obtained with the Sulfo-NHS-SS-biotin reagent. Indeed, with this strategy, we were able to identify 8 nitrated BSA peptides from only 1 mg of total protein, meaning that the use of direct capture with aldehyde-agarose beads resulted in both greatly improved specificity/selectivity, as compared with the NHS-mediated tagging method.

### Identification of nitrated proteins and their nitration sites in untreated and peroxynitrite-treated rat brain homogenate

The fully optimized ANSID protocol is depicted in Figure 1 and proceeds as follows: (1) digestion of proteins with trypsin, (2) dimethylation of Lys- and N-terminal amines on peptides with formaldehyde/DMAB, (3) reduction of aromatic nitro groups to amino groups with hemin/DTT, (4) capture of amino-aromatic amino acid-containing peptides with aldehyde-agarose beads, (5) peptide elution with TFA, and (6) analysis by LC-MS/MS. In order to test the utility of this method for the discovery of nitrated proteins and their nitration sites in a complex biological mixture, we performed the ANSID on rat brain homogenates that were either untreated or nitrated by treatment with 250 μM or 1 mM ONOO^−^. For each group, the ANSID was performed on six replicate rat brain homogenates, and all of the nitrated peptides that were identified in at least two of the six replicates are listed in Table 1 (untreated), Table 2 (250 μM ONOO^−^) and Supplementary Table 1 (1 mM ONOO^−^).

**Table 1:** **Nitrated peptides identified in untreated rat brain homogenate (Spectrum Mill score of ≥ 9, corresponding to a false positive rate of < 2%)**.

**Protein**	**Accession #**	**References**	**Peptide**	**References**	**Samples present**	**Average score**
14-3-3 protein gamma	P61983	Sultana et al., 2007	(K)TAFDDAIAELDTLNEDSYK(D)^*^		2	15.60
Actin, cytoplasmic	P60711	Zhang et al., 2007; Ghesquière et al., 2009	(R)kDLYANTVLSGGTTMYPGIADR(M)	Zhang et al., 2007; Ghesquière et al., 2009	3	20.24
Seipin	Q5FVJ6		(R)SVmLHYR(S)		6	12.46

* *Dimetylated lysine residues are denoted by lower case “k” and nitrated tyrosine residues are denoted by red upper case Y*.

**Table 2:** **Nitrated peptides identified in 250 μM peroxynitrite-treated rat brain homogenate (Spectrum Mill score of ≥ 9, corresponding to a false positive rate of < 2%)**.

**Protein**	**Accession #**	**References**	**Peptide**	**References**	**Samples present**	**Average score**
14-3-3 protein beta/alpha	P35213	Zhang et al., 2007; Ghesquière et al., 2009	(K)TAFDEAIAELDTLNEESYk(D)^*^	Zhang et al., 2007; Ghesquière et al., 2009	2	15.60
14-3-3 protein epsilon	P62260	Zhang et al., 2007; Ghesquière et al., 2009	(K)AAFDDAIAELDTLSEESYk(D)	Zhang et al., 2007; Ghesquière et al., 2009	3	20.24
14-3-3 protein theta	P68255	Zhang et al., 2007	(K)TAFDEAIAELDTLNEDSYk(D)	Zhang et al., 2007	6	12.46
14-3-3 protein zeta/delta	P63102	Zhang et al., 2007; Stevens et al., 2008	(K)TAFDEAIAELDTLSEESYk(D)	Zhang et al., 2007	6	18.41
			(R)YLAEVAAGDDk(K)	Stevens et al., 2008; Ghesquière et al., 2009	2	12.74
Glutamate dehydrogenase 1, mitochondrial	P10860	Aulak et al., 2001; Reed et al., 2009	(K)VYEGSILEADcDILIPAASEk(Q)		5	15.16
Hemoglobin subunit alpha-1/2	P01946	Li et al., 2011	(K)TYFSHIDVSPGSAQVk(A)	Li et al., 2011	6	16.83
			(K)IGGHGGEYGEEALQR(M)		4	16.22
Hemoglobin subunit beta-1	P02091	Zhang et al., 2007; Li et al., 2011	(R)YFDSFGDLSSASAImGNPk(V)		5	13.12
Myelin basic protein S	P02688	Zhang et al., 2007; Stevens et al., 2008	(K)YLATASTMDHAR(H)	Zhang et al., 2007; Stevens et al., 2008	5	15.92
			(R)TTHYGSLPQk(S)	Zhang et al., 2007	3	14.13
Phosphoglycerate mutase 1	P25113	Zhang et al., 2007; Ghesquière et al., 2009; Reed et al., 2009	(R)FSGWYDADLSPAGHEEAk(R)		5	17.87
Pyruvate kinase isozymes M1/M2	P11980	Kanski et al., 2005b; Ghesquière et al., 2009	(K)ITLDNAYMEk(C) (R)LNFSHGTHEYHAETIk(N)	Kanski et al., 2005b; Ghesquière et al., 2009	5	10.64
					3	19.75
Synapsin-2	Q63537		(K)VENHYDFQDIASVVALTQTYATAEPFIDAk(Y)		2	10.05
Tubulin alpha-1B chain	P68370	Zhang et al., 2007; Ghesquière et al., 2009	(R)FDGALNVDLTEFQTNLVPYPR(I)	Zhang et al., 2007	2	10.99
			(R)LSVDYGk(K)	Ghesquière et al., 2009	2	9.69
Tubulin beta-2A chain	P85108	Zhang et al., 2007; Stevens et al., 2008	(K)GHYTEGAELVDSVLDVVR(K)	Stevens et al., 2008	6	15.24
			(K)LTTPTyGDLNHLVSATMSGVTTcLR(F)		2	13.5

* *Dimetylated lysine residues and carbimethylated cystene residues are denoted by lower case “k” and “c”, respectively. Nitrated tyrosine residues are denoted by a red upper case Y*.

**Figure 3:**
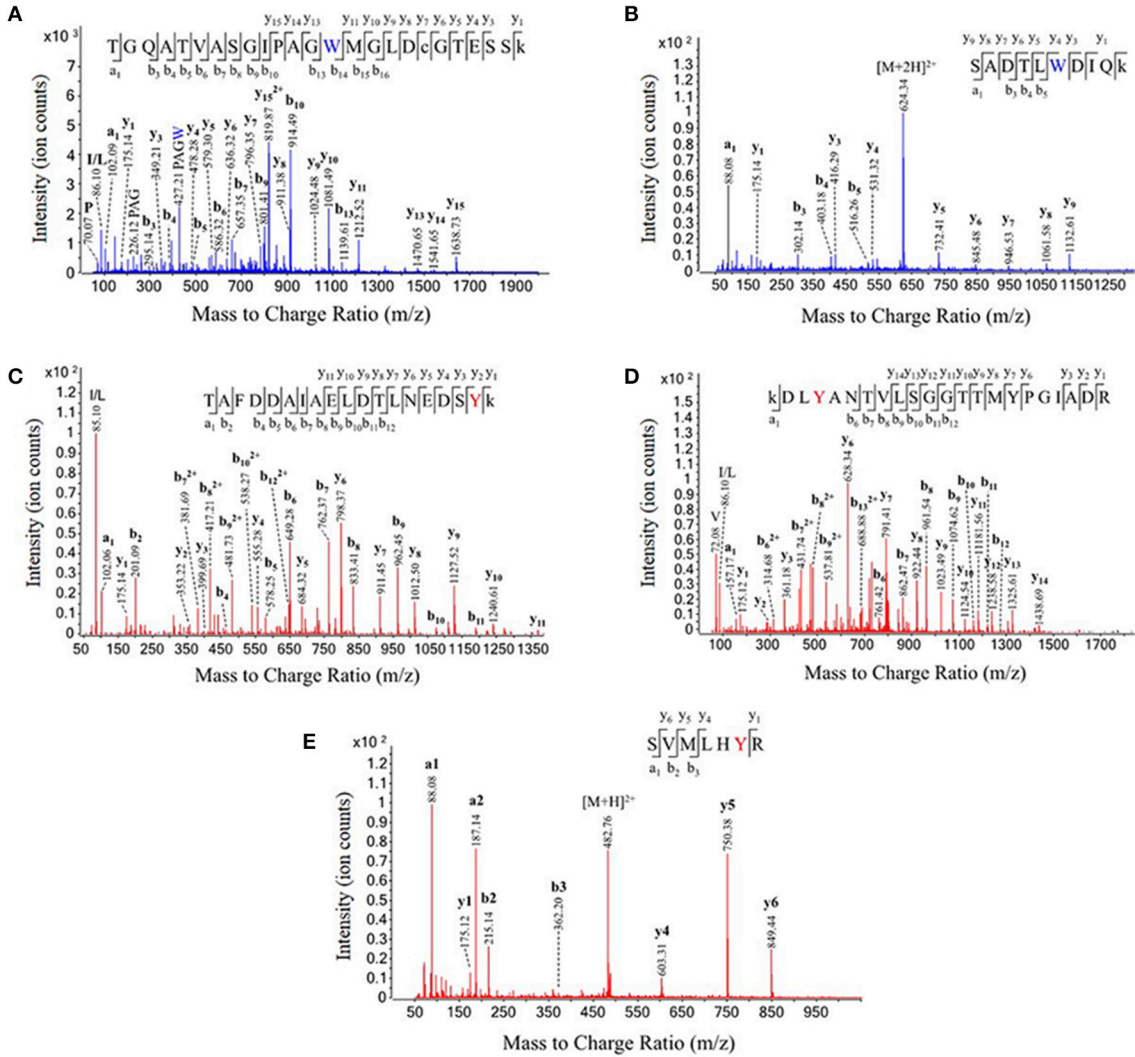
**Validation of tryptophan nitration sites in peroxynitrite-treated rat brain homogenate and tyrosine nitration sites in untreated rat brain homogenate**. The labeled MS/MS spectra from five nitrated peptides. The identities of each of the two tryptophan nitrated peptides discovered in the 1 mM ONOO^−^-treated brain homogenates **(A,B)** and each of the three tyrosine nitrated peptides discovered in the untreated rat brain homogenates **(C–E)** were manually validated using their MS/MS fragmentation data. Dimethylated lysines and carbamidomethylated cysteines are represented by under case letters, and nitration sites are represented by colored letters.

In total, we identified 3 nitrated peptides from 3 proteins that were identified in at least two of the six replicates from the untreated rat brain homogenates, 19 nitrated peptides from 13 proteins in 250 μM ONOO^−^-treated rat brain homogenates and 244 nitrated peptides from 145 proteins in 1 mM ONOO^−^-treated rat brain homogenates. While 99% of the nitrated peptides identified in the 1 mM ONOO^−^-treated rat brain homogenates were nitrated on a Tyr residue, we also identified 2 peptides that were nitrated on a Trp residue. These Trp-nitrated peptides, as well as the 3 Tyr-nitrated peptides identified in untreated rat brain homogenates, were all manually validated through analysis of their MS/MS spectra and their identity was confirmed (Figure 3). To our knowledge, these results mark the first time that an in-solution enrichment method has identified Trp-nitrated proteins in a biologically-complex protein mixture.

### Modification of the ANSID for relative quantification of nitrated peptides

Because nitration levels may be predicted to vary with disease progression and in response to therapeutic treatments, the capability to monitor changes in nitration levels of specific proteins and sites will be critical for determining the significance of aromatic ring nitration for disease progression/regression. However, MS is not an inherently quantitative technique, and the number of ion counts per pmol of any identified peptide will be highly dependent on relative ionization and competition for ionization (ion suppression) by co-eluting species (Ong and Mann, 2005). One way to overcome this limitation is to perform isotope-labeling, where one sample is labeled with a reagent that contains the common light isotope of hydrogen and/or carbon, and the other samples is labeled with the identical reagent, but containing a heavy isotope (i.e., deuterium and/or carbon-13). This heavy/light labeling is followed by sample pooling and concurrent further processing. Finally, samples are analyzed by LC-MS/MS for ratiometric comparison of the isotopic abundances of all the observed peptides (Gevaert et al., 2008). With this strategy, differentially-labeled peptides will elute from the LC column at identical retention times and MS detection sensitivities, but distinguishable from one another based on distinct mass-to-charge (m/z) ratios.

Using this isotopic labeling approach, we created a ratiometric quantitative ANSID technique by utilizing heavy and light formaldehyde for dimethylation of the Lys- and N-terminal amino groups during the amine-blocking step. The heavy formaldehyde was >98% enriched with one carbon-13 and two deuterium atoms, giving it a total mass difference of +3 Da over the standard light formaldehyde, which was >98% enriched with one carbon-12 atom and two hydrogen atoms. Since the dimethylation step used in our method ultimately results in the incorporation of two carbon atom and four hydrogen atoms from formaldehyde per labeled amino acid, dimethylation with heavy formaldehyde predictably results in a mass shift of +6 Da for tryptic peptides containing a C-terminal Arg residue (where only the N-terminal amine is modified) and +12 Da for peptides containing a C-terminal Lys residue (where both the N-terminal amine and amino group in Lys are modified).

In order to test this strategy for quantitative ANSID, we compared the relative levels of nitrated peptides detected in undiluted rat brain homogenates vs. homogenates diluted to varying extents with exogenously nitrated brain homogenates. Specifically, nitrated peptides were extracted from undiluted rat brain homogenate exposed to 1 mM ONOO^−^, compared to with untreated rat brain homogenate diluted 1:1, 1:4, 1:9, or 1:19 with the nitrated peptide homogenate. For this quantitative ANSID procedure, the non-nitrated sample was dimethylated using light formaldehyde, while the nitrated samples were dimethylated using heavy formaldehyde. Afterwards, the dimethylation reactions were quenched by incubation with glycine, and each undiluted sample was pooled with one of the four diluted samples for processing and analysis by LC-MS/MS.

**Figure 4:**
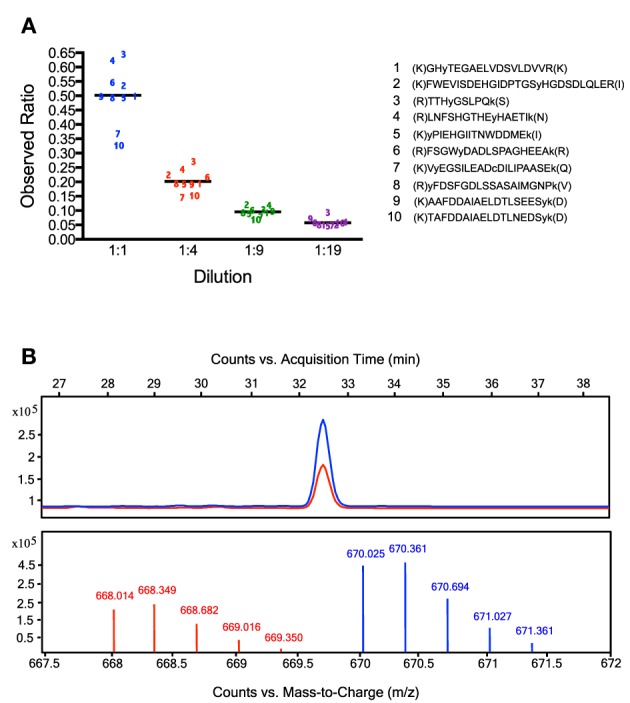
**Relative quantification of nitrated rat brain peptides using dimethylation with heavy and light formaldehyde**. Results from experiments comparing the relative quantities of nitrated peptides extracted from undiluted vs. diluted samples of nitrated rat brain homogenate. Panel **(A)** depicts the observed vs. expected ratios in each dilution group of the 10 most abundant nitrated peptides. Each observed ratio value is the mean value from three replicate experiments. The average observed ratios are 0.502 for the 1:1 dilution, 0.202 for the 1:4 dilution, 0.096 for the 1:9 dilution, and 0.058 for the 1:19 dilution. In Panel **(B)**, the chromatograms from the purified heavy and light versions of the nitrated rat brain peptide GHyTEGAELVDSVLDVVR are shown. As this peptide is triply charged and contains just one dimethylation site (the N-terminus), the m/z difference between the heavy and light peptides is +2 (6 Da/3 charges). Though these equivalent peptides co-elute, they clearly separate from one another on the m/z axis.

To assess the linearity for quantification in this experiment, we considered the 10 most abundant nitrated peptides and determined the relative quantities (comparing ion counts) for light vs. heavy versions of these peptides in each of the pooled mixtures (*n* = 4). As shown in Figure 4A, the observed ratios of heavy vs. light nitrated peptides were found to be in accord with the predicted ratios for each dilution, with an average observed heavy:light ratio of 0.502 for the 1:1 dilution (0.326–0.644 range), 0.202 for the 1:4 dilution (0.146–0.271 range), 0.096 for the 1:9 dilution (0.068–0.120 range), and 0.058 for the 1:19 dilution (0.046–0.093 range). Furthermore, as shown in Figure 4B, it is clear that while the equivalent heavy and light peptides co-elute, there is enough separation on the m/z axis to easily differentiate between them, even for peptides with only one dimethylation group, such as the rat brain peptide GHyTEGAELVDSVLDVVR.

## Discussion

The lack of a robust method for enriching and identifying nitrated proteins from endogenously nitrated tissues represents a significant obstacle in the study of how protein nitration impacts human physiology and pathology. Although the global levels of nitration in a given disease state can be easily quantified with applications such as high-performance liquid chromatography with electrochemical detection (HPLC-ECD) (Nuriel et al., 2008; Zhou et al., 2011) and multiple-reaction monitoring (MRM) MS (Ishii et al., 2006; Danielson et al., 2009), it is difficult to investigate the true impact of protein nitration without the knowledge of which specific proteins become nitrated in various settings and on which specific amino acid residues. In this report, we describe a new method for identifying both exogenously and endogenously nitrated proteins and their sites of nitration. We anticipate that application of this method will provide a significant step forward in efforts to specifically identify and provide a relative quantification of nitration sites in settings of health and disease. The ANSID approach proceeds through a simple and straightforward protocol (Figure 1) that results in the enrichment of nitrated peptides from complex proteolytic mixtures for identification by LC-MS/MS. Although similar methods have been previously reported (Nikov et al., 2003; Zhang et al., 2007; Abello et al., 2010; Tsumoto et al., 2010; Dremina et al., 2011; Prokai-Tatrai et al., 2011; Guo et al., 2012), the utility of these methods has been essentially limited to the identification of small amounts of nitrated proteins from exogenously nitrated samples. In addition, the majority of these methods could not be employed for relative nitrated peptide quantification and could only identify peptides/proteins containing 3-NT residues, and not those nitrated on other aromatic amino acids.

In developing this ANSID method, we hypothesized that the limited effectiveness of the previously described methods arise from inefficiencies of the reagents and methodologies utilized. Therefore, we focused our efforts on optimizing each individual step in the enrichment protocol to achieve maximal selectivity and yield. Over the course of our optimization efforts, we identified multiple enhancements relative to the in-solution enrichment strategies that had been previously employed. Indeed, for the amine-blocking step, we found that reductive dimethylation with formaldehyde and DMAB allowed for the rapid and efficient modification of primary amines and, unlike acetylation, this reaction did not remove their positive charge at acidic pH and thereby enabled sensitive MS detection (Figure 2A). For the nitro-group reduction step, we found that heating samples in the presence of hemin-agarose beads and DTT resulted in essentially complete reduction of 3-NT to 3-AT and did not result in the generation of significant side-products, as was the case for reduction with sodium dithionite (Figure 2B). And for the capture of the resulting amino-aromatic containing peptides, we found that solid-phase capture with aldehyde-agarose beads was more effective at enriching 3-AT-containing peptides from an NO2-BSA/rat brain homogenate mixture, compared to affinity-tagging with Sulfo-NHS-SS-Biotin, followed by a streptavidin-sepharose bead capture (Figure 2C). Importantly, all of these optimized strategies utilize inexpensive, commercially available reagents.

It is interesting to note that the solid-phase capture strategy utilized in the ANSID was also employed to improve upon the SNOSID method for identifying S-nitrosylated proteins/sites, allowing for endogenously S-nitrosylated proteins to be identified in large numbers for the first time (Doulias et al., 2010). It should also be noted that a similar reductive dimethylation strategy was employed for the amine-blocking step in an in-solution effort to identifying nitrated proteins and their sites of nitration by Prokai-Tatrai et al. (2011). Abello et al. reported the use of a similar heme/DTT reduction of 3-NT residues in their in-solution enrichment method (Abello et al., 2010). The ANSID approach described herein assembles what we have found to be the most efficacious and optimized procedural steps for detection of protein nitration sites and additionally provides for their relative quantification.

Once optimized, we utilized the ANSID method to identify nitrated proteins in exogenously nitrated rat brain homogenates. In 1 mM ONOO^−^-treated rat brain homogenates, we discovered 244 nitrated peptides from 145 proteins (Supplementary Table 1), with 6 of these proteins being isoforms of 14-3-3. These identified proteins are involved in a wide range of cellular functions, including cell signaling, energy metabolism, protein transport, and host defense. Out of the 145 total nitrated proteins identified in the 1 mM ONOO^−^-treated homogenates, 55% were previously identified as nitrated, and of the 244 nitration sites identified, 18% were previously recognized as targets of nitration. These prior reports confirm both the validity of the ANSID method and its potential for discovery, since many of the nitrated proteins and nitration sites we identified had previously been identified by other groups, as well as the value of the method, since a large number of previously undiscovered proteins and sites were identified.

We also identified 19 nitrated peptides from 13 proteins that were present in at least two out of six 250 μM ONOO^−^-treated rat brain homogenate samples (Table 2), with 4 of these 13 proteins being isoforms of 14-3-3. Of these 19 nitrated peptides, 18 of them were also present in the 1 mM ONOO^−^ group, suggesting that these 18 nitration sites have a particular sensitivity to ONOO^−^, as compared to those found in the other abundant proteins found to be nitrated only with higher concentrations of ONOO^−^.

Perhaps most importantly, we identified three nitrated peptides from three proteins in untreated rat brain homogenates, demonstrating that the ANSID method is capable of identifying endogenously nitrated proteins and their sites of nitration. These results also demonstrate that aromatic nitration occurs not only during pathological conditions, but can also occur in the setting of normal physiology, a possibility that has been the subject of considerable debate among protein nitration investigators (Turko and Murad, 2002; Schopfer et al., 2003; Gow et al., 2004). The three proteins identified as being endogenously nitrated in this study were 14-3-3 protein gamma, actin, and seipin. Although little is known about the function of seipin, it is highly expressed in the brain, and inheritance of mutated forms of the seipin gene have been shown to cause a number of human disorders, including Berardinelli-Seip congenital lipodystrophy, autosomal-dominant distal hereditary motor neuropathy type V and Silver syndrome. Thus, the revelation that seipin can be nitrated under physiological conditions may have biological relevance. The fact that nitrated 14-3-3 protein was observed in our untreated brains may also have biological significance. 14-3-3 is a regulatory protein that has been shown to bind over 100 proteins, including many proteins involved in cell signaling via phosphorylation (Mhawech, 2005). Given our observation that 14-3-3 protein becomes nitrated during physiological conditions, it is worth investigating whether 14-3-3 protein may reside in cells in proximity to one or more NOS isoforms, placing it in proximity to endogenously formed NO.

Importantly, these results also allowed for the identification of proteins nitrated on Trp residues. It is worth noting that two of the three Trp-nitrated proteins that were identified in the ONOO^−^-treated rat brain homogenates (L-lactate dehydrogenase B chain and phosphoglycerate kinase 1) were also identified using a 2D-Gel electrophoresis technique employing an anti-6-nitrotryptophan antibody (Uda et al., 2012), although this prior report did not allow for the identification of the specifically modified Trp residues as accomplished using the ANSID method. We anticipate that the ANSID method will greatly aid in the investigation of these relatively understudied Trp-nitrated protein modifications.

We adapted the ANSID method to allow for the relative quantification of the nitrated peptides by performing a dimethylation step with heavy vs. light formaldehyde (Figure 4). Because the stable-isotope labeling that results from this quantitative ANSID approach results in the replacement of at least four hydrogen atoms with at least four deuterium atoms per peptide, there was some concern that chromatographic isotope effects might diminish the accuracy of the relative quantification performed in this method (Gevaert et al., 2008). Notably, deuterium atoms are slightly more hydrophilic than hydrogen atoms, which means that deuterium-containing peptides could potentially elute from the stationary phase slightly earlier than their hydrogen-containing counterparts during LC-MS/MS analysis. This could in-turn cause discrepancies in the ionization efficiencies of these related peptides, mainly due to matrix interference by co-eluting substances (Taylor, 2005). However, an analysis of the retention times of the heavy and light versions of the nitrated rat brain peptide depicted in Figure 4B (GHyTEGAELVDSVLDVVR) reveals no differences in elution patterns, as the elution of heavy GHyTEGAELVDSVLDVVR occurred at a retention time (32.112–32.887 min) indistinguishable from the light peptide. This observation that differential dimethylation with heavy and light formaldehyde does not result in significant chromatographic isotope effects has been reported previously (Ji et al., 2005), including in the report by Guo et al., which also utilized isotope-coded reductive dimethylation for the relative quantification of nitrated proteins (Guo et al., 2012).

In conclusion, the ANSID approach described here offers a new method for the unbiased identification and quantification of nitrated proteins and their sites of nitration in biologically-complex mixtures of proteins. The method provides several significant enhancements over the previously reported in-solution enrichment strategies for nitration site identification, including relative quantification, increased sensitivity and the ability to identify Trp-nitrated proteins and sites. While further advances may be necessary to comprehensively identify a majority of the nitration sites present in endogenous tissues, we expect that the ANSID method will greatly aid researchers in their study of protein nitration and the potential roles of this posttransltional modification in disease pathogenesis and normal physiology.

## Author contributions

This study was designed and managed by TN and SG. Experiments were performed by TN, JW, EM, and NB. Mass spectrometry was performed by TN, JW, and YM. Further data interpretation and analysis was performed by TN, JW, and YM. The paper was written by TN and SG.

### Conflict of interest statement

The authors declare that the research was conducted in the absence of any commercial or financial relationships that could be construed as a potential conflict of interest.
