# Supplementary material for: ANSID: A Solid-Phase Proteomic Approach for Identification and Relative Quantification of Aromatic Nitration Sites
Source: Front Chem. 2016 Jan 7;3:70. doi: 10.3389/fchem.2015.00070 (PMC4703760; doi:10.3389/fchem.2015.00070)
Supplement: Supplementary file 1 [file Table1.DOCX]

Supplementary Table 1. Nitrated peptides identified in 1 mM peroxynitrite-treated rat brain homogenate (Spectrum Mill score of ≥ 9, corresponding to a false positive rate of < 2%).

| **Protein** | **Accession #** | **Refs.** | **Peptide** | **Refs.** | **Samples Present** | **Average Score** |
| --- | --- | --- | --- | --- | --- | --- |
| 14-3-3 protein beta/alpha | [P35213](http://www.uniprot.org/uniprot/P35213) | ([Ghesquiere et al., 2009](#_ENREF_7); [Zhang et al., 2007](#_ENREF_32)) | (K)TAFDEAIAELDTLNEES**Y**k(D) | ([Ghesquiere et al., 2009](#_ENREF_7); [Zhang et al., 2007](#_ENREF_32)) | 6 | 19.18 |
|  |  |  | (R)**Y**LSEVASGDNk(Q) |  | 2 | 14.77 |
| 14-3-3 protein epsilon | [P62260](http://www.uniprot.org/uniprot/P62260) | ([Ghesquiere et al., 2009](#_ENREF_7); [Zhang et al., 2007](#_ENREF_32)) | (K)AAFDDAIAELDTLSEES**Y**k(D) | ([Ghesquiere et al., 2009](#_ENREF_7); [Zhang et al., 2007](#_ENREF_32)) | 6 | 19.11 |
| 14-3-3 protein eta | [P68511](http://www.uniprot.org/uniprot/P68511) |  | (K)QAFDDAIAELDTLNEDS**Y**k(D) |  | 5 | 19.81 |
|  |  |  | (R)**Y**LAEVASGEk(K) |  | 3 | 14.34 |
| 14-3-3 protein gamma | [P61983](http://www.uniprot.org/uniprot/P61983) | ([Sultana et al., 2007](#_ENREF_24)) | (K)ELEAVcQDVLSLLDN**Y**LIk(N) |  | 6 | 19.44 |
|  |  |  | (R)LGLALNYSVFY**Y**EIQNAPEQAcHLAk(T) |  | 5 | 21.55 |
|  |  |  | (R)**Y**LAEVATGEk(R) |  | 5 | 15.29 |
| 14-3-3 protein theta | [P68255](http://www.uniprot.org/uniprot/P68255) | ([Zhang et al., 2007](#_ENREF_32)) | (K)TAFDEAIAELDTLNEDS**Y**k(D) | ([Zhang et al., 2007](#_ENREF_32)) | 6 | 19.13 |
| 14-3-3 protein zeta/delta | [P63102](http://www.uniprot.org/uniprot/P63102) | ([Stevens et al., 2008](#_ENREF_22); [Zhang et al., 2007](#_ENREF_32)) | (K)TAFDEAIAELDTLSEES**Y**k(D) | ([Zhang et al., 2007](#_ENREF_32)) | 6 | 18.83 |
|  |  |  | (R)**Y**LAEVAAGDDk(K) | ([Ghesquiere et al., 2009](#_ENREF_7); [Stevens et al., 2008](#_ENREF_22)) | 5 | 14.10 |
| Acetyl-CoA acetyltransferase, mitochondrial | [P17764](http://www.uniprot.org/uniprot/P17764) | ([Zhang et al., 2007](#_ENREF_32)) | (R)IAAFADAAVDPIDFPLAPA**Y**AVPk(V) |  | 6 | 21.70 |
| Aconitate hydratase, mitochondrial | [Q9ER34](http://www.uniprot.org/uniprot/Q9ER34) | ([Vattemi et al., 2011](#_ENREF_29)) | (R)SDFDPGQDT**Y**QHPPk(D) |  | 5 | 16.14 |
|  |  |  | (K)VAMSHFEPSE**Y**IR(Y) |  | 3 | 18.16 |
| Actin, cytoplasmic | [P60711](http://www.uniprot.org/uniprot/P60711) | ([Ghesquiere et al., 2009](#_ENREF_7); [Kanski et al., 2005b](#_ENREF_12); [Sacksteder et al., 2006](#_ENREF_20); [Tyther et al., 2007](#_ENREF_27); [Zhang et al., 2007](#_ENREF_32)) | (K)DLYANTVLSGGTTM**Y**PGIADR(M) |  | 6 | 15.28 |
|  |  |  | (R)G**Y**SFTTTAER(E) | ([Ghesquiere et al., 2009](#_ENREF_7); [Zhang et al., 2007](#_ENREF_32)) | 6 | 16.54 |
|  |  |  | (K)IWHHTF**Y**NELR(V) |  | 6 | 16.62 |
|  |  |  | (K)S**Y**ELPDGQVITIGNER(F) | ([Ghesquiere et al., 2009](#_ENREF_7)) | 6 | 16.50 |
|  |  |  | (K)QE**Y**DESGPSIVHR(K) | ([Ghesquiere et al., 2009](#_ENREF_7)) | 5 | 22.28 |
|  |  |  | (K)**Y**PIEHGIITNWDDMEk(I) |  | 5 | 12.81 |
|  |  |  | (R)TTGIVMDSGDGVTHTVPIYEG**Y**ALPHAILR(L) |  | 2 | 17.94 |
|  |  |  | (R)DLTD**Y**LMk(I) | ([Ghesquiere et al., 2009](#_ENREF_7)) | 2 | 11.35 |
| Actin-related protein 2 | [Q5M7U6](http://www.uniprot.org/uniprot/Q5M7U6) |  | (R)SmLEVN**Y**PMENGIVR(N) |  | 2 | 11.14 |
| Actin-related protein 3 | [Q4V7C7](http://www.uniprot.org/uniprot/Q4V7C7) | ([Ghesquiere et al., 2009](#_ENREF_7)) | (R)DIT**Y**FIQQLLR(D) |  | 6 | 17.92 |
| Adapter molecule crk | [Q63768](http://www.uniprot.org/uniprot/Q63768) |  | (K)IH**Y**LDTTTLIEPVSR(S) |  | 3 | 14.13 |
| ADP-ribosylation factor 3 | [P61206](http://www.uniprot.org/uniprot/P61206) |  | (R)H**Y**FQNTQGLIFVVDSNDR(E) |  | 6 | 14.14 |
|  |  |  | (K)LGEIVTTIPTIGFNVETVE**Y**kN) |  | 6 | 16.91 |
|  |  |  | (R)NW**Y**IQATcATSGDGLYEGLDWLANQLk(N) |  | 5 | 19.23 |
| ADP-ribosylation factor GTPase-activating protein 1 | [Q62848](http://www.uniprot.org/uniprot/Q62848) |  | (K)AFEDWLNDDLGS**Y**QGAQENR(Y) |  | 3 | 16.95 |
| Alcohol dehydrogenase [NADP+] | [P51635](http://www.uniprot.org/uniprot/P51635) | ([Crow et al., 1995](#_ENREF_4)) | (R)DAGHPLYPFNDP**Y**(-) |  | 3 | 11.79 |
|  |  |  | (R)HIDcASV**Y**GNETEIGEALk(E) |  | 2 | 15.39 |
| Alpha-enolase | [P04764](http://www.uniprot.org/uniprot/P04764) | ([Castegna et al., 2003](#_ENREF_3); [Ghesquiere et al., 2009](#_ENREF_7); [Reed et al., 2009b](#_ENREF_19); [Sultana et al., 2007](#_ENREF_24); [Xiao et al., 2005](#_ENREF_31)) | (R)AAVPSGASTGI**Y**EALELR(D) | ([Ghesquiere et al., 2009](#_ENREF_7)) | 6 | 16.07 |
|  |  |  | (K)D**Y**PVVSIEDPFDQDDWDAWQk(F) |  | 5 | 23.77 |
|  |  |  | (K)AGYTDQVVIGMDVAASEF**Y**R(A) |  | 3 | 17.46 |
|  |  |  | (R)GNPTVEVDL**Y**TAk(G) |  | 3 | 14.74 |
| Alpha-synuclein | [P37377](http://www.uniprot.org/uniprot/P37377) | ([Giasson et al., 2000](#_ENREF_9); [Zhang et al., 2007](#_ENREF_32)) | (K)EGVL**Y**VGSk(T) | ([Giasson et al., 2000](#_ENREF_9); [Zhang et al., 2007](#_ENREF_32)) | 4 | 14.55 |
|  |  |  | (K)GEEGYPQEGILEDMPVDPSSEAYEMPSEEG**Y**QDYEPEA(-) | ([Giasson et al., 2000](#_ENREF_9)) | 3 | 13.93 |
| Amphiphysin | [O08838](http://www.uniprot.org/uniprot/O08838) |  | (K)LTESLHEVYEPDW**Y**GR(E) |  | 6 | 17.39 |
|  |  |  | (K)DEQFEE**Y**VQNFk(R) |  | 4 | 16.42 |
| Amyloid beta A4 protein | [P08592](http://www.uniprot.org/uniprot/P08592) |  | (K)**Y**LETPGDENEHAHFQk(A) |  | 2 | 15.56 |
| Aspartate aminotransferase, cytoplasmic | [P13221](http://www.uniprot.org/uniprot/P13221) | ([Miyagi et al., 2002](#_ENREF_16); [Sacksteder et al., 2006](#_ENREF_20); [Zhang et al., 2007](#_ENREF_32)) | (R)FLFPFFDSA**Y**QGFASGDLEk(D) |  | 5 | 15.87 |
|  |  |  | (K)IANDHSLNHE**Y**LPILGLAEFR(S) |  | 2 | 15.68 |
| ATP synthase-coupling factor 6, mitochondrial | [P21571](http://www.uniprot.org/uniprot/P21571) |  | (R)LASGGPVDTGPE**Y**QQEVDR(E) |  | 3 | 18.23 |
| ATP synthase subunit beta, mitochondrial | [P10719](http://www.uniprot.org/uniprot/P10719) | ([Ghesquiere et al., 2009](#_ENREF_7); [Vattemi et al., 2011](#_ENREF_29); [Zhang et al., 2007](#_ENREF_32)) | (R)AIAELGI**Y**PAVDPLDSTSR(I) | ([Ghesquiere et al., 2009](#_ENREF_7); [Zhang et al., 2007](#_ENREF_32)) | 6 | 17.37 |
|  |  |  | (R)EGNDL**Y**HEMIESGVINLk(D) | ([Zhang et al., 2007](#_ENREF_32)) | 6 | 15.81 |
|  |  |  | (R)IPSAVG**Y**QPTLATDMGTMQER(I) |  | 6 | 17.51 |
|  |  |  | (R)IMDPNIVGSEH**Y**DVAR(G) | ([Ghesquiere et al., 2009](#_ENREF_7); [Zhang et al., 2007](#_ENREF_32)) | 5 | 19.28 |
| Brevican core protein | [P55068](http://www.uniprot.org/uniprot/P55068) |  | (R)E**Y**QWIGLNDR(T) |  | 5 | 12.58 |
| Calbindin | [P07171](http://www.uniprot.org/uniprot/P07171) |  | (K)AFEL**Y**DQDGNG**Y**IDENELDALLk(D) |  | 6 | 15.93 |
| Calcium-dependent secretion activator 1 | [Q62717](http://www.uniprot.org/uniprot/Q62717) |  | (R)**Y**VDLMESSIAQSIHR(G) |  | 2 | 12.68 |
| Calcyclin-binding protein | [Q6AYK6](http://www.uniprot.org/uniprot/Q6AYK6) |  | (K)EkPS**Y**DTEADPSEGLMNVLk(K) |  | 3 | 13.13 |
| Calmodulin | [P62161](http://www.uniprot.org/uniprot/P62161) | ([Smallwood et al., 2003](#_ENREF_21)) | (K)DGNG**Y**ISAAELR(H) | ([Smallwood et al., 2003](#_ENREF_21)) | 6 | 17.72 |
|  |  |  | (R)EADIDGDGQVN**Y**EEFVQMmTAk(-) |  | 2 | 19.42 |
| Calreticulin | [P18418](http://www.uniprot.org/uniprot/P18418) | ([Ghesquiere et al., 2009](#_ENREF_7)) | (K)SGTIFDNFLITNDEA**Y**AEEFGNETWGVTk(A) |  | 6 | 23.15 |
| Calumenin | [O35783](http://www.uniprot.org/uniprot/O35783) | ([Ghesquiere et al., 2009](#_ENREF_7)) | (K)DWILPSD**Y**DHAEAEAR(H) |  | 2 | 15.31 |
| Carbonic anhydrase 2 | [P27139](http://www.uniprot.org/uniprot/P27139) | ([Ghosh et al., 2006](#_ENREF_8); [Sultana et al., 2006](#_ENREF_23)) | (K)**Y**AAELHLVHWNTk(Y) |  | 6 | 17.10 |
| Casein kinase II subunit beta | [P67874](http://www.uniprot.org/uniprot/P67874) |  | (R)V**Y**cENQPMLPIGLSDIPGEAMVk(L) |  | 2 | 10.86 |
| Chloride intracellular channel protein 4 | [Q9Z0W7](http://www.uniprot.org/uniprot/Q9Z0W7) |  | (K)EVEIA**Y**SDVAk(R) |  | 3 | 15.34 |
| Citrate synthase, | [Q8VHF5](http://www.uniprot.org/uniprot/Q8VHF5) | ([Ghesquiere et al., 2009](#_ENREF_7); [Zhang et al., 2007](#_ENREF_32)) | (K)LVAQL**Y**k(I) |  | 5 | 12.12 |
| mitochondrial |  |  |  |  |  |  |
| Clathrin coat assembly protein AP180 | [Q05140](http://www.uniprot.org/uniprot/Q05140) |  | (K)HLD**Y**LIQATNETNVNIPQMADTLFER(A) |  | 2 | 15.17 |
| Clathrin heavy chain 1 | [P11442](http://www.uniprot.org/uniprot/P11442) | ([Ghesquiere et al., 2009](#_ENREF_7); [Gokulrangan et al., 2007](#_ENREF_10); [Suzuki et al., 2005](#_ENREF_25); [Zhang et al., 2007](#_ENREF_32)) | (R)ALEHFTDL**Y**DIk(R) |  | 2 | 15.69 |
|  |  |  | (R)FQSVPAQPGQTSPLLQYFGILLDQGQLNk**Y**ESLELcRPVLQQGR(K) |  | 2 | 17.07 |
|  |  |  | (R)TSIDA**Y**DNFDNISLAQR(L) |  | 2 | 14.69 |
| Cofilin-1 | [P45592](http://www.uniprot.org/uniprot/P45592) | ([Ghesquiere et al., 2009](#_ENREF_7); [Gokulrangan et al., 2007](#_ENREF_10); [Zhang et al., 2007](#_ENREF_32)) | (K)EILVGDVGQTVDDP**Y**TTFVk(M) | ([Ghesquiere et al., 2009](#_ENREF_7); [Zhang et al., 2007](#_ENREF_32)) | 6 | 19.04 |
| Complexin-2 | [P84087](http://www.uniprot.org/uniprot/P84087) |  | (K)**Y**LPGPLQDMFk(K) |  | 3 | 9.84 |
| Coronin-1A | [Q91ZN1](http://www.uniprot.org/uniprot/Q91ZN1) |  | (K)SDLFQEDL**Y**PPTAGPDPALTAEEWLSGR(D) |  | 2 | 19.73 |
|  |  |  | (R)YFEITSEAPFLH**Y**LSMFSSk(E) |  | 3 | 15.95 |
| Creatine kinase B-type | [P07335](http://www.uniprot.org/uniprot/P07335) | ([Zhang et al., 2007](#_ENREF_32)) | (K)cTPSGFTLDDAIQTGVDNPGHP**Y**ImTVGAVAGDEESYDVFk(D) |  | 2 | 15.81 |
| Creatine kinase U-type, mitochondrial | [P25809](http://www.uniprot.org/uniprot/P25809) | ([Kanski et al., 2005a](#_ENREF_11); [Lee et al., 2009](#_ENREF_14); [Zhang et al., 2007](#_ENREF_32)) | (K)TVGMVAGDEET**Y**EVFAELFDPVIQER(H) | ([Zhang et al., 2007](#_ENREF_32)) | 6 | 15.91 |
|  |  |  | (R)L**Y**PPSAEYPDLR(K) |  | 2 | 14.82 |
| Cytosolic non-specific dipeptidase | [Q6Q0N1](http://www.uniprot.org/uniprot/Q6Q0N1) |  | (K)ILIPGINDAVAPVTDEEHEL**Y**DHIDFDMEEFAk(D) |  | 6 | 18.92 |
| Dihydropyrimidinase-related protein 1 | [Q62950](http://www.uniprot.org/uniprot/Q62950) | ([Sacksteder et al., 2006](#_ENREF_20)) | (R)Gm**Y**DGPV**Y**EVPATPk(H) |  | 6 | 12.56 |
|  |  |  | (R)IINDDQSF**Y**ADVYLEDGLIk(Q) |  | 6 | 17.27 |
|  |  |  | (K)STVE**Y**NIFEGMEcHGSPLVVISQGk(I) |  | 4 | 19.03 |
| Dihydropyrimidinase-related protein 2 | [P47942](http://www.uniprot.org/uniprot/P47942) | ([Miyagi et al., 2002](#_ENREF_16); [Sultana et al., 2007](#_ENREF_24); [Zhang et al., 2007](#_ENREF_32)) | (K)THNSALE**Y**NIFEGMEcR(G) |  | 6 | 12.46 |
|  |  |  | (K)IVNDDQSF**Y**ADIYMEDGLIk(Q) |  | 6 | 16.49 |
|  |  |  | (R)GL**Y**DGPVcEVSVTPk(T) |  | 5 | 16.45 |
|  |  |  | (K)GTVV**Y**GEPITASLGTDGSHYWSk(N) |  | 2 | 18.27 |
| Drebrin-like protein | [Q9JHL4](http://www.uniprot.org/uniprot/Q9JHL4) |  | (K)ESScFQDVGPQAPVGSV**Y**Qk(T) |  | 2 | 14.67 |
| Dynamin-1 | [P21575](http://www.uniprot.org/uniprot/P21575) | ([Sacksteder et al., 2006](#_ENREF_20); [Zhang et al., 2007](#_ENREF_32)) | (K)EFIFSELLANL**Y**ScGDQNTLMEESAEQAQR(R) |  | 6 | 22.36 |
|  |  |  | (R)IEGSGDQIDT**Y**ELSGGAR(I) | ([Zhang et al., 2007](#_ENREF_32)) | 5 | 15.78 |
|  |  |  | (R)RPLVLQLVNSTTE**Y**AEFLHck(G) |  | 4 | 20.44 |
| Dynamin-2 | [P39052](http://www.uniprot.org/uniprot/P39052) |  | (K)VPVGDQPPDIE**Y**QIk(D) |  | 2 | 16.14 |
| Dynamin-3 | [Q08877](http://www.uniprot.org/uniprot/Q08877) |  | (K)DFINSELLAQL**Y**SSEDQNTLMEESVEQAQR(R) |  | 2 | 12.99 |
| EH domain-containing protein 3 | [Q8R491](http://www.uniprot.org/uniprot/Q8R491) |  | (K)GGAFEGTLQGPFGHG**Y**GEGAGEGIDDAEWVVAR(D) |  | 4 | 15.48 |
| Electron transfer flavoprotein subunit | [P13803](http://www.uniprot.org/uniprot/P13803) | ([Zhang et al., 2007](#_ENREF_32)) | (K)DPEAPIFQVAD**Y**GIVADLFk(V) |  | 2 | 14.24 |
| alpha, mitochondrial |  |  |  |  |  |  |
| Elongation factor 1-alpha 1 | [P62630](http://www.uniprot.org/uniprot/P62630) | ([Fan et al., 2011](#_ENREF_6); [Ghesquiere et al., 2009](#_ENREF_7)) | (R)LPLQDV**Y**k(I) | ([Ghesquiere et al., 2009](#_ENREF_7)) | 5 | 15.43 |
|  |  |  | (K)SGDAAIVDMVPGkPMcVESFSD**Y**PPLGR(F) |  | 3 | 16.60 |
|  |  |  | (K)IG**Y**NPDTVAFVPISGWNGDNMLEPSANMPWFk(G) |  | 2 | 14.63 |
| Elongation factor Tu, mitochondrial | [P85834](http://www.uniprot.org/uniprot/P85834) | ([Kanski et al., 2005a](#_ENREF_11)) | (K)LLDAVDT**Y**IPVPTR(D) |  | 3 | 13.43 |
| Endophilin-A1 | [O35179](http://www.uniprot.org/uniprot/O35179) |  | (K)GPG**Y**PQAEALLAEAMLk(F) |  | 6 | 15.36 |
|  |  |  | (R)AL**Y**DFEPENEGELGFk(E) |  | 5 | 19.71 |
|  |  |  | (R)LDFD**Y**k(K) |  | 2 | 9.51 |
| Endophilin-A2 | [O35964](http://www.uniprot.org/uniprot/O35964) |  | (K)AL**Y**DFEPENDGELGFR(E) |  | 2 | 18.48 |
|  |  |  | (R)LDFD**Y**k(K) |  | 2 | 9.51 |
| Endoplasmic reticulum resident protein 29 | [P52555](http://www.uniprot.org/uniprot/P52555) |  | (R)LAENSASSDDLLVAEVGISD**Y**GDk(L) |  | 6 | 18.19 |
| Endoplasmin | [Q66HD0](http://www.uniprot.org/uniprot/Q66HD0) | ([Ghesquiere et al., 2009](#_ENREF_7)) | (R)GLFDE**Y**GSk(K) |  | 5 | 13.72 |
| Enoyl-CoA hydratase domain-containing protein 1 | [Q6AYG5](http://www.uniprot.org/uniprot/Q6AYG5) |  | (R)EL**Y**LEEALQNER(D) |  | 2 | 12.38 |
| Eukaryotic initiation factor 4A-II | [Q5RKI1](http://www.uniprot.org/uniprot/Q5RKI1) | ([Ghesquiere et al., 2009](#_ENREF_7)) | (K)G**Y**DVIAQAQSGTGk(T) | ([Ghesquiere et al., 2009](#_ENREF_7)) | 4 | 20.46 |
| Ezrin | [P31977](http://www.uniprot.org/uniprot/P31977) | ([Fan et al., 2011](#_ENREF_6); [Ghesquiere et al., 2009](#_ENREF_7)) | (K)F**Y**PEDVADELIQDITQk(L) |  | 5 | 14.75 |
| Fructose-bisphosphate aldolase A | [P05065](http://www.uniprot.org/uniprot/P05065) | ([Aulak et al., 2001](#_ENREF_1); [Koeck et al., 2004](#_ENREF_13); [Miyagi et al., 2002](#_ENREF_16); [Reed et al., 2009b](#_ENREF_19); [Stevens et al., 2008](#_ENREF_22)) | (K)**Y**TPSGQSGAAASESLFISNHA**Y**(-) | ([Koeck et al., 2004](#_ENREF_13)) | 6 | 15.73 |
| Fumarate hydratase, mitochondrial | [P14408](http://www.uniprot.org/uniprot/P14408) | ([Ghesquiere et al., 2009](#_ENREF_7); [Zhang et al., 2007](#_ENREF_32)) | (R)IE**Y**DTFGELk(V) | ([Ghesquiere et al., 2009](#_ENREF_7)) | 3 | 10.03 |
|  |  |  | (K)TAIELG**Y**LTAEQFDEWVkPk(D) |  | 2 | 14.42 |
| Glucose-regulated protein 78 | [P06761](http://www.uniprot.org/uniprot/P06761) | ([Sacksteder et al., 2006](#_ENREF_20); [Zhang et al., 2007](#_ENREF_32)) | (K)L**Y**GSGGPPPTGEEDTSEkDEL(-) |  | 3 | 12.67 |
| Glutamate decarboxylase 2 | [Q05683](http://www.uniprot.org/uniprot/Q05683) |  | (K)VIDFH**Y**PNELLQEYNWELADQPQNLEEILTHcQTTLk(Y) |  | 2 | 17.73 |
| Glutamate dehydrogenase 1, mitochondrial | [P10860](http://www.uniprot.org/uniprot/P10860) | ([Aulak et al., 2001](#_ENREF_1); [Reed et al., 2009b](#_ENREF_19)) | (R)DSN**Y**HLLMSVQESLER(K) |  | 3 | 12.25 |
|  |  |  | (K)V**Y**EGSILEADcDILIPAASEk(Q) |  | 6 | 18.64 |
| Glutathione S-transferase alpha-3 | [P04904](http://www.uniprot.org/uniprot/P04904) |  | (R)ALIDMYAEGVADLDEIVLHYP**Y**IPPGEk(E) |  | 5 | 16.74 |
| Glutathione S-transferase Mu 1 | [P04905](http://www.uniprot.org/uniprot/P04905) | ([Aulak et al., 2001](#_ENREF_1)) | (R)YAMGDAPD**Y**DR(S) |  | 6 | 11.95 |
|  |  |  | (R)LLLE**Y**TDSSYEEk(R) |  | 5 | 14.94 |
|  |  |  | (R)Lc**Y**NPDFEk(L) |  | 5 | 12.58 |
| Glyceraldehyde-3-phosphate dehydrogenase | [P04797](http://www.uniprot.org/uniprot/P04797) | ([Aulak et al., 2001](#_ENREF_1); [Buchczyk et al., 2003](#_ENREF_2); [Ghosh et al., 2006](#_ENREF_8); [Gokulrangan et al., 2007](#_ENREF_10); [Kanski et al., 2005a](#_ENREF_11); [Kanski et al., 2005b](#_ENREF_12); [Miyagi et al., 2002](#_ENREF_16); [Sultana et al., 2006](#_ENREF_23); [Tyther et al., 2007](#_ENREF_27); [Xiao et al., 2005](#_ENREF_31)) | (K)LISW**Y**DNE**Y**G**Y**SNR(V) |  | 6 | 15.15 |
|  |  |  | (K)GILG**Y**TEDQVVScDFNSNSHSSTFDAGAGIALNDNFVk(L) |  | 5 | 16.63 |
|  |  |  | (K)VDIVAINDPFIDLN**Y**MV**Y**MFQ**Y**DSTHGk(F) |  | 5 | 14.26 |
| Glycine cleavage system H protein, mitochondrial | [Q5I0P2](http://www.uniprot.org/uniprot/Q5I0P2) |  | (K)DGIGTVGISNFAQEALGDVV**Y**cSLPEVGTk(L) |  | 5 | 18.10 |
| Glycogen phosphorylase, brain form (Fragment) | [P53534](http://www.uniprot.org/uniprot/P53534) | ([Kanski et al., 2005b](#_ENREF_12)) | (K)DFNVGD**Y**IEAVLDR(N) |  | 6 | 19.38 |
|  |  |  | (K)DVVNMLM**Y**HDR(F) |  | 2 | 15.39 |
|  |  |  | (R)VL**Y**PNDNFFEGk(E) |  | 6 | 12.18 |
| Growth factor receptor-bound protein 2 | [P62994](http://www.uniprot.org/uniprot/P62994) | ([Ghesquiere et al., 2009](#_ENREF_7)) | (R)DIEQVPQQPT**Y**VQALFDFDPQEDGELGFR(R) |  | 2 | 13.99 |
| GTP-binding nuclear protein Ran | [P62828](http://www.uniprot.org/uniprot/P62828) | ([Ghesquiere et al., 2009](#_ENREF_7)) | (K)LIGDPNLEFVAMPALAPPEVVMDPALAAQ**Y**EHDLEVAQTTALPDEDDDL(-) |  | 6 | 11.90 |
| Guanine nucleotide-binding protein G(I)/G(S)/G(T) subunit beta-1 | [P54311](http://www.uniprot.org/uniprot/P54311) |  | (K)I**Y**AMHWGTDSR(L) |  | 3 | 16.30 |
| Heat shock 10 kDa protein, mitochondrial | [P26772](http://www.uniprot.org/uniprot/P26772) | ([Ghesquiere et al., 2009](#_ENREF_7)) | (K)VLLPE**Y**GGTk(V) |  | 6 | 14.77 |
| Heat shock 60 kDa protein, mitochondrial | [P63039](http://www.uniprot.org/uniprot/P63039) | ([Ghesquiere et al., 2009](#_ENREF_7)) | (K)ILQSSSEVG**Y**DAMLGDFVNMVEk(G) | ([Ghesquiere et al., 2009](#_ENREF_7)) | 6 | 17.02 |
|  |  |  | (R)IQEITEQLDITTSE**Y**Ek(E) |  | 5 | 17.13 |
| Heat shock 70 kDa protein 4 | [O88600](http://www.uniprot.org/uniprot/O88600) | ([Reed et al., 2009a](#_ENREF_18); [Zhang et al., 2007](#_ENREF_32)) ([Ghesquiere et al., 2009](#_ENREF_7)) | (R)AGGIETIANE**Y**SDR(C) |  | 4 | 13.94 |
| Heat shock protein HSP 90-alpha | [P82995](http://www.uniprot.org/uniprot/P82995) | ([Ghesquiere et al., 2009](#_ENREF_7); [Xiao et al., 2005](#_ENREF_31); [Zhang et al., 2007](#_ENREF_32)) | (R)GFEVVYMTEPIDE**Y**cVQQLk(E) |  | 4 | 19.77 |
|  |  |  | (K)IR**Y**ESLTDPSk(L) | ([Ghesquiere et al., 2009](#_ENREF_7)) | 4 | 12.88 |
|  |  |  | (K)HGLEVIYMIEPIDE**Y**cVQQLk(E) |  | 2 | 18.05 |
|  |  |  | (K)HI**Y**FITGETk(D) | ([Ghesquiere et al., 2009](#_ENREF_7)) | 2 | 12.39 |
|  |  |  | (R)Y**Y**TSASGDEmVSLk(D) |  | 2 | 13.24 |
| Hemoglobin subunit alpha-1/2 | [P01946](http://www.uniprot.org/uniprot/P01946) | ([Li et al., 2011](#_ENREF_15)) | (K)IGGHGGE**Y**GEEALQR(M) |  | 6 | 17.32 |
|  |  |  | (K)T**Y**FSHIDVSPGSAQVk(A) | ([Li et al., 2011](#_ENREF_15)) | 4 | 17.33 |
| Hemoglobin subunit beta-1 | [P02091](http://www.uniprot.org/uniprot/P02091) | ([Li et al., 2011](#_ENREF_15); [Zhang et al., 2007](#_ENREF_32)) | (K)EFTPcAQAAFQkVVAGVASALAHk**Y**H(-) |  | 6 | 17.26 |
|  |  |  | (R)LLVV**Y**PWTQR(Y) | ([Li et al., 2011](#_ENREF_15)) | 6 | 14.17 |
|  |  |  | (R)**Y**FDSFGDLSSASAImGNPk(V) |  | 6 | 17.69 |
|  |  |  | (K)VVAGVASALAHk**Y**H(-) | ([Li et al., 2011](#_ENREF_15); [Zhang et al., 2007](#_ENREF_32)) | 5 | 16.42 |
| Heterogeneous nuclear ribonucleoprotein K | [P61980](http://www.uniprot.org/uniprot/P61980) | ([Ghesquiere et al., 2009](#_ENREF_7)) | (R)GS**Y**GDLGGPIITTQVTIPk(D) |  | 2 | 14.47 |
| High mobility group  protein B1 | [P63159](http://www.uniprot.org/uniprot/P63159) |  | (K)RPPSAFFLFcSE**Y**RPk(I) |  | 2 | 13.66 |
| Hsp90 co-chaperone | [Q63692](http://www.uniprot.org/uniprot/Q63692) |  | (R)LGPGGLDPVEV**Y**ESLPEELQk(C) |  | 5 | 13.62 |
| Cdc37 |  |  |  |  |  |  |
| Hyaluronan and proteoglycan link protein 1 | [P03994](http://www.uniprot.org/uniprot/P03994) |  | (R)QAcLDQDAVIASFDQL**Y**DAWR(G) |  | 3 | 21.32 |
| Inosine triphosphate pyrophosphatase | [D3ZW55](http://www.uniprot.org/uniprot/D3ZW55) |  | (K)IDLPE**Y**QGEPDEISIQk(C) |  | 3 | 12.02 |
| Isocitrate dehydrogenase [NAD] subunit alpha, mitochondrial | [Q99NA5](http://www.uniprot.org/uniprot/Q99NA5) |  | (R)ENTEGE**Y**SGIEHVIVDGVVQSIk(L) |  | 6 | 17.85 |
| Isocitrate dehydrogenase [NAD] subunit beta, mitochondrial | [Q68FX0](http://www.uniprot.org/uniprot/Q68FX0) |  | (R)DMGG**Y**STTTDFIk(S) |  | 5 | 18.59 |
|  |  |  | (R)HLNLE**Y**HSSMIADAVk(K) |  | 5 | 15.01 |
|  |  |  | (K)LGDGLFLQccEEVAEL**Y**Pk(I) |  | 2 | 13.46 |
| Isocitrate dehydrogenase [NAD] subunit gamma 1, mitochondrial | [P41565](http://www.uniprot.org/uniprot/P41565) |  | (R)ENTEGE**Y**SSLEHESVAGVVESLk(I) |  | 2 | 17.85 |
| L-lactate dehydrogenase A chain | [P04642](http://www.uniprot.org/uniprot/P04642) |  | (K)QVVDSA**Y**EVIk(L) |  | 5 | 15.07 |
|  |  |  | (K)LLIVSNPVDILT**Y**VAWk(I) |  | 3 | 10.59 |
| L-lactate dehydrogenase B chain | [P42123](http://www.uniprot.org/uniprot/P42123) | ([Ghesquiere et al., 2009](#_ENREF_7); [Uda et al., 2012](#_ENREF_28)) | (K)SADTL**W**DIQk(D) |  | 6 | 18.24 |
|  |  |  | (K)MVVDSA**Y**EVIk(L) | ([Ghesquiere et al., 2009](#_ENREF_7)) | 2 | 11.92 |
| Lactoylglutathione lyase | [Q6P7Q4](http://www.uniprot.org/uniprot/Q6P7Q4) |  | (K)GLAFVQDPDG**Y**WIEILNPNk(M) |  | 3 | 10.53 |
| Lon protease homolog, mitochondrial | [Q924S5](http://www.uniprot.org/uniprot/Q924S5) | ([Ghesquiere et al., 2009](#_ENREF_7)) | (R)GYQGDPSSALLELLDPEQNANFLDH**Y**LDVPVDLSk(V) |  | 3 | 14.73 |
|  |  |  | (R)VVDNPI**Y**LSDMGAALTGAESHELQDVLEETNILk(R) |  | 2 | 16.08 |
| Malate dehydrogenase, cytoplasmic | [O88989](http://www.uniprot.org/uniprot/O88989) | ([Gokulrangan et al., 2007](#_ENREF_10); [Kanski et al., 2005a](#_ENREF_11); [Tyther et al., 2007](#_ENREF_27)) | (K)EVGV**Y**EALk(D) |  | 5 | 13.54 |
| Malate dehydrogenase, mitochondrial | [P04636](http://www.uniprot.org/uniprot/P04636) | ([Ghesquiere et al., 2009](#_ENREF_7); [Stevens et al., 2008](#_ENREF_22); [Zhang et al., 2007](#_ENREF_32)) | (R)LTL**Y**DIAHTPGVAADLSHIETR(A) | ([Stevens et al., 2008](#_ENREF_22)) | 5 | 21.43 |
|  |  |  | (K)G**Y**LGPEQLPDcLk(G) | ([Stevens et al., 2008](#_ENREF_22)) | 4 | 16.29 |
| Microtubule-associated protein 4 | [Q5M7W5](http://www.uniprot.org/uniprot/Q5M7W5) | ([Ghesquiere et al., 2009](#_ENREF_7)) | (R)DFMAALEAEP**Y**DDIVGETVEk(T) |  | 2 | 14.84 |
| Microtubule-associated protein 6 | [Q63560](http://www.uniprot.org/uniprot/Q63560) |  | (R)SE**Y**QPSDAPFER(E) |  | 2 | 12.20 |
| Microtubule-associated protein RP/EB family member 1 | [Q66HR2](http://www.uniprot.org/uniprot/Q66HR2) |  | (R)IVDILYATDEGFVIPDEGGPQEEQEE**Y**(-) |  | 2 | 12.68 |
| Mitogen-activated protein kinase 3 | [P21708](http://www.uniprot.org/uniprot/P21708) |  | (R)DVYIVQDLMETDL**Y**k(L) |  | 6 | 12.50 |
|  |  |  | (R)IEVEQALAHPYLEQ**YY**DPSDEPIAEAPFk(F) |  | 4 | 18.18 |
|  |  |  | (R)ITVEEALAHPYLEQY**Y**DPTDEPVAEEPFTFDMELDDLPk(E) |  | 2 | 19.31 |
| Myelin basic protein S | [P02688](http://www.uniprot.org/uniprot/P02688) | ([Stevens et al., 2008](#_ENREF_22); [Zhang et al., 2007](#_ENREF_32)) | (R)FSWGAEGQkPGFG**Y**GGR(A) |  | 5 | 15.23 |
|  |  |  | (K)GA**Y**DAQGTLSk(I) |  | 4 | 16.67 |
|  |  |  | (K)**Y**LATASTMDHAR(H) | ([Stevens et al., 2008](#_ENREF_22); [Zhang et al., 2007](#_ENREF_32)) | 3 | 15.28 |
|  |  |  | (R)TTH**Y**GSLPQk(S) | ([Zhang et al., 2007](#_ENREF_32)) | 2 | 13.74 |
| Myc box-dependent-interacting protein 1 | [O08839](http://www.uniprot.org/uniprot/O08839) |  | (K)LSEcLQEV**Y**EPEWPGR(D) |  | 4 | 15.64 |
| NADH dehydrogenase [ubiquinone] 1 alpha subcomplex subunit 10, mitochondrial | [Q561S0](http://www.uniprot.org/uniprot/Q561S0) | ([Li et al., 2011](#_ENREF_15)) | (K)VVEDIE**Y**LNYNk(G) |  | 2 | 11.80 |
| Neuroendocrine protein 7B2 | [P27682](http://www.uniprot.org/uniprot/P27682) |  | (K)DFSEDQG**Y**PDPPNPcPLGk(T) |  | 2 | 18.95 |
| PC4 and SFRS1-interacting protein | [Q812D1](http://www.uniprot.org/uniprot/Q812D1) |  | (K)G**Y**PHWPAR(V) |  | 5 | 15.76 |
| Peptidyl-prolyl cis-trans isomerase FKBP4 | [Q9QVC8](http://www.uniprot.org/uniprot/Q9QVC8) | ([Xiao et al., 2005](#_ENREF_31)) | (K)IVSWLE**Y**ESSFSGEEMQk(V) |  | 2 | 15.67 |
| Peroxiredoxin-2 | [P35704](http://www.uniprot.org/uniprot/P35704) | ([Reed et al., 2009b](#_ENREF_19)) | (R)LVQAFQ**Y**TDEHGEVcPAGWkPGSDTIkPNVDDSk(E) |  | 5 | 20.52 |
| Phosphatidylethanolamine-binding protein 1 | [P31044](http://www.uniprot.org/uniprot/P31044) | ([Ghesquiere et al., 2009](#_ENREF_7)) | (K)GNDISSGTVLSE**Y**VGSGPPk(D) |  | 5 | 16.11 |
|  |  |  | (R)VD**Y**GGVTVDELGk(V) |  | 5 | 16.81 |
| Phosphatidylinositol transfer protein alpha isoform | [P16446](http://www.uniprot.org/uniprot/P16446) |  | (R)TVITNE**Y**Mk(E) |  | 2 | 11.24 |
| Phosphoglucomutase-1 | [P38652](http://www.uniprot.org/uniprot/P38652) |  | (K)VFQGNAN**Y**AENFIQSIVSTVEPALR(Q) |  | 2 | 10.78 |
| Phosphoglycerate kinase 1 | [P16617](http://www.uniprot.org/uniprot/P16617) | ([Ghesquiere et al., 2009](#_ENREF_7); [Gokulrangan et al., 2007](#_ENREF_10); [Reed et al., 2009a](#_ENREF_18); [Uda et al., 2012](#_ENREF_28); [Zhang et al., 2007](#_ENREF_32)) | (K)TGQATVASGIPAG**W**MGLDcGTESSk(K) |  | 5 | 21.39 |
|  |  |  | (K)VLNNMEIGTSL**Y**DEEGAk(I) |  | 5 | 16.56 |
|  |  |  | (K)ELN**Y**FAk(A) | ([Ghesquiere et al., 2009](#_ENREF_7)) | 4 | 13.23 |
| Phosphoglycerate mutase 1 | [P25113](http://www.uniprot.org/uniprot/P25113) | ([Ghesquiere et al., 2009](#_ENREF_7); [Reed et al., 2009b](#_ENREF_19); [Zhang et al., 2007](#_ENREF_32)) | (R)FSGW**Y**DADLSPAGHEEAk(R) |  | 5 | 21.96 |
|  |  |  | (R)DAG**Y**EFDIcFTSVQk(R) |  | 4 | 16.41 |
|  |  |  | (R)SYDVPPPPMEPDHPF**Y**SNISk(D) |  | 2 | 13.49 |
| Profilin-2 | [Q9EPC6](http://www.uniprot.org/uniprot/Q9EPC6) | ([Ghesquiere et al., 2009](#_ENREF_7)) | (K)**Y**VWAATAGGVFQSITPAEIDVIIGk(D) |  | 6 | 14.31 |
| Protein CDV3 homolog | [Q5XIM5](http://www.uniprot.org/uniprot/Q5XIM5) | ([Ghesquiere et al., 2009](#_ENREF_7)) | (K)TPQGPPEI**Y**SDTQFPSLQSTAk(H) |  | 6 | 16.11 |
| Protein FAM54B | [Q5XII9](http://www.uniprot.org/uniprot/Q5XII9) | ([Ghesquiere et al., 2009](#_ENREF_7)) | (K)DVPPVPTLADIAWIAADEEET**Y**AR(V) |  | 2 | 14.36 |
| Protein kinase C and casein kinase substrate in neurons protein 1 | [Q9Z0W5](http://www.uniprot.org/uniprot/Q9Z0W5) | ([Zhang et al., 2007](#_ENREF_32)) | (R)ALYD**Y**DGQEQDELSFk(A) |  | 6 | 16.40 |
|  |  |  | (R)LDSGQLGLYPAN**Y**VEAI(-) | ([Zhang et al., 2007](#_ENREF_32)) | 6 | 14.35 |
| Protein kinase C and | [Q9QY17](http://www.uniprot.org/uniprot/Q9QY17) |  | (R)AL**Y**DYEGQEHDELSFk(A) |  | 4 | 15.49 |
| casein kinase substrate in neurons protein 2 |  |  |  |  |  |  |
| Polyubiquitin-C | [Q63429](http://www.uniprot.org/uniprot/Q63429) |  | (R)TLSD**Y**NIQk(E) |  | 6 | 13.66 |
| Pyruvate dehydrogenase E1 component subunit beta, mitochondrial | [P49432](http://www.uniprot.org/uniprot/P49432) | ([Vattemi et al., 2011](#_ENREF_29)) | (K)VFLLGEEVAQ**Y**DGA**Y**k(V) |  | 6 | 17.82 |
| Pyruvate kinase isozymes M1/M2 | [P11980](http://www.uniprot.org/uniprot/P11980) | ([Ghesquiere et al., 2009](#_ENREF_7); [Kanski et al., 2005b](#_ENREF_12)) | (R)AATESFASDPIL**Y**RPVAVALDTk(G) |  | 6 | 17.90 |
|  |  |  | (K)GAD**Y**LVTEVENGGSLGSk(K) |  | 6 | 19.28 |
|  |  |  | (K)GD**Y**PLEAVR(M) |  | 6 | 16.18 |
|  |  |  | (K)ITLDNA**Y**MEk(C) | ([Ghesquiere et al., 2009](#_ENREF_7); [Kanski et al., 2005b](#_ENREF_12)) | 6 | 12.75 |
|  |  |  | (K)I**Y**VDDGLISLQVk(E) |  | 6 | 15.43 |
|  |  |  | (R)LNFSHGTHE**Y**HAETIk(N) |  | 6 | 17.83 |
| Rab GDP dissociation inhibitor alpha | [P50398](http://www.uniprot.org/uniprot/P50398) | ([Ghesquiere et al., 2009](#_ENREF_7); [Zhang et al., 2007](#_ENREF_32)) | (K)**Y**IAIASTTVETAEPEkEVEPALELLEPIDQk(F) |  | 6 | 15.22 |
|  |  |  | (R)NPY**Y**GGESSSITPLEELYk(R) |  | 2 | 10.43 |
| Ras-related protein Rab-7a | [P09527](http://www.uniprot.org/uniprot/P09527) | ([Ghesquiere et al., 2009](#_ENREF_7)) | (K)QETEVEL**Y**NEFPEPIk(L) |  | 2 | 15.26 |
| Receptor-type tyrosine-protein phosphatase zeta | [Q62656](http://www.uniprot.org/uniprot/Q62656) |  | (K)**Y**SDQLIVDMPTEDAELDLFPELIGTEEIIk(E) |  | 5 | 16.78 |
|  |  |  | (K)YSDQLIVDMPTEDAELDLFPELIGTEEIIkEEN**Y**Gk(G) |  | 2 | 19.06 |
| Reticulocalbin-2 | [Q62703](http://www.uniprot.org/uniprot/Q62703) |  | (K)LSEEEILENQDLFLTSEATD**Y**GR(Q) |  | 3 | 18.72 |
| Secretogranin-2 | [P10362](http://www.uniprot.org/uniprot/P10362) |  | (R)TNEIVEEQ**Y**TPQSLATLESVFQELGk(L) |  | 3 | 16.54 |
| Septin-7 | [Q9WVC0](http://www.uniprot.org/uniprot/Q9WVC0) |  | (K)STLINSLFLTDLYSPE**Y**PGPSHR(I) |  | 3 | 20.86 |
| Serine-threonine kinase receptor-associated protein | [Q5XIG8](http://www.uniprot.org/uniprot/Q5XIG8) | ([Ghesquiere et al., 2009](#_ENREF_7)) | (K)IGFPETAEEELEEIASENSDSI**Y**SSTPEVk(A) |  | 4 | 16.90 |
| Serine/threonine-protein phosphatase 2A catalytic subunit alpha isoform | [P63331](http://www.uniprot.org/uniprot/P63331) |  | (K)**Y**SFLQFDPAPR(R) |  | 2 | 13.97 |
| Serine/threonine-protein phosphatase 2B catalytic subunit alpha isoform | [P63329](http://www.uniprot.org/uniprot/P63329) |  | (R)FkEPPA**Y**GPMcDILWSDPLEDFGNEk(T) |  | 5 | 21.76 |
| Serotransferrin | [P12346](http://www.uniprot.org/uniprot/P12346) | ([Li et al., 2011](#_ENREF_15); [Piroddi et al., 2011](#_ENREF_17)) | (R)kPVDQYEDc**Y**LAR(I) |  | 4 | 16.84 |
| Serum albumin | [P02770](http://www.uniprot.org/uniprot/P02770) | ([Fan et al., 2011](#_ENREF_6); [Ghosh et al., 2006](#_ENREF_8); [Li et al., 2011](#_ENREF_15); [Miyagi et al., 2002](#_ENREF_16); [Piroddi et al., 2011](#_ENREF_17); [Turko et al., 2003](#_ENREF_26); [Tyther et al., 2007](#_ENREF_27)) | (R)LPcVED**Y**LSAILNR(L) |  | 6 | 14.79 |
|  |  |  | (K)DDNPNLPPFQRPEAEAMcTSFQENPTSFLGH**Y**LHEVAR(R) |  | 2 | 15.55 |
| Spliceosome RNA helicase Ddx39b | [Q63413](http://www.uniprot.org/uniprot/Q63413) |  | (R)FEVNISELPDEIDISS**Y**IEQTR(-) |  | 3 | 20.13 |
| Sulfated glycoprotein 1 | [P10960](http://www.uniprot.org/uniprot/P10960) |  | (K)EVVDS**Y**LPVILDMIk(G) |  | 2 | 17.87 |
| Synapsin-1 | [P09951](http://www.uniprot.org/uniprot/P09951) | ([Reed et al., 2009a](#_ENREF_18)) | (K)EmLSSTT**Y**PVVVk(M) |  | 3 | 17.71 |
|  |  |  | (K)LGTEEFPLIDQTF**Y**PNHk(E) |  | 3 | 19.19 |
| Synapsin-2 | [Q63537](http://www.uniprot.org/uniprot/Q63537) |  | (K)VENH**Y**DFQDIASVVALTQTYATAEPFIDAk(Y) |  | 6 | 17.03 |
| Synaptojanin-1 | [Q62910](http://www.uniprot.org/uniprot/Q62910) |  | (R)TSPcQSPTAPE**Y**SAPSLPIRPSR(A) |  | 5 | 19.57 |
| Synaptophysin | [P07825](http://www.uniprot.org/uniprot/P07825) | ([Di Stasi et al., 1999](#_ENREF_5)) | (R)LHQV**Y**FDAPScVk(G) |  | 3 | 16.25 |
| Synaptotagmin-1 | [P21707](http://www.uniprot.org/uniprot/P21707) | ([Vrljic et al., 2011](#_ENREF_30)) | (K)VQVVVTVLD**Y**Dk(I) | ([Vrljic et al., 2011](#_ENREF_30)) | 2 | 15.32 |
| Syntaxin-binding protein 1 | [P61765](http://www.uniprot.org/uniprot/P61765) |  | (K)MPQ**Y**Qk(E) |  | 4 | 11.05 |
|  |  |  | (K)**Y**STHLHLAEDcMk(H) |  | 3 | 15.58 |
| Thiosulfate sulfurtransferase | [P24329](http://www.uniprot.org/uniprot/P24329) | ([Aulak et al., 2001](#_ENREF_1)) | (R)**Y**LGTQPEPDAVGLDSGHIR(G) |  | 4 | 18.42 |
| Transforming protein RhoA | [P61589](http://www.uniprot.org/uniprot/P61589) |  | (K)DQFPEVYVPTVFEN**Y**VADIEVDGk(Q) |  | 6 | 21.56 |
| Transgelin-3 | [P37805](http://www.uniprot.org/uniprot/P37805) |  | (K)LINSL**Y**PPGQEPIPk(I) |  | 3 | 14.01 |
| Transketolase | [P50137](http://www.uniprot.org/uniprot/P50137) | ([Aulak et al., 2001](#_ENREF_1); [Ghosh et al., 2006](#_ENREF_8); [Zhang et al., 2007](#_ENREF_32)) | (K)NMAEQIIQEI**Y**SQVQSk(K) | ([Aulak et al., 2001](#_ENREF_1)) | 5 | 17.59 |
| Tubulin alpha-1B chain | [P68370](http://www.uniprot.org/uniprot/P68370) | ([Ghesquiere et al., 2009](#_ENREF_7); [Zhang et al., 2007](#_ENREF_32)) | (K)A**Y**HEQLSVAEITNAcFEPANQMVk(C) |  | 6 | 15.14 |
|  |  |  | (R)FDGALNVDLTEFQTNLVP**Y**PR(I) | ([Zhang et al., 2007](#_ENREF_32)) | 6 | 17.07 |
|  |  |  | (R)IHFPLAT**Y**APVISAEk(A) | ([Zhang et al., 2007](#_ENREF_32)) | 5 | 14.85 |
|  |  |  | (K)VGIN**Y**QPPTVVPGGDLAR(V) | ([Ghesquiere et al., 2009](#_ENREF_7); [Zhang et al., 2007](#_ENREF_32)) | 5 | 16.98 |
|  |  |  | (R)AFVHW**Y**VGEGmEEGEFSEAR(E) |  | 4 | 18.54 |
|  |  |  | (K)LEFSIYPAPQVSTAVVEPYNSILTTHTTLEHSDcAFMVDNEAI**Y**DIcR(R) |  | 2 | 18.29 |
|  |  |  | (R)LSVD**Y**Gk(K) | ([Ghesquiere et al., 2009](#_ENREF_7)) | 2 | 13.40 |
| Tubulin beta-2A chain | [P85108](http://www.uniprot.org/uniprot/P85108) | ([Stevens et al., 2008](#_ENREF_22); [Zhang et al., 2007](#_ENREF_32)) | (K)GH**Y**TEGAELVDSVLDVVR(K) | ([Stevens et al., 2008](#_ENREF_22)) | 6 | 15.85 |
|  |  |  | (K)LATPT**Y**GDLNHLVSATMSGVTTSLR(F) | ([Stevens et al., 2008](#_ENREF_22)) | 6 | 13.32 |
|  |  |  | (K)FWEVISDEHGIDPTGS**Y**HGDSDLQLER(I) |  | 5 | 19.08 |
|  |  |  | (K)NSS**Y**FVEWIPNNVk(T) |  | 5 | 16.14 |
|  |  |  | (K)VSDTVVEPYNATLSVHQLVENTDET**Y**SIDNEALYDIcFR(T) |  | 2 | 17.54 |
| Ubiquitin-like modifier-activating enzyme 1 | [Q5U300](http://www.uniprot.org/uniprot/Q5U300) |  | (R)I**Y**DDDFFQNLDGVANALDNVDAR(M) |  | 2 | 15.09 |
| Ubiquitin-conjugating enzyme E2 N | [Q9EQX9](http://www.uniprot.org/uniprot/Q9EQX9) | ([Ghesquiere et al., 2009](#_ENREF_7); [Li et al., 2011](#_ENREF_15)) | (R)**Y**FHVVIAGPQDSPFEGGTFk(L) |  | 2 | 18.41 |
| UV excision repair protein RAD23 homolog B | [Q4KMA2](http://www.uniprot.org/uniprot/Q4KMA2) |  | (R)SNLFEDATSALVTGQS**Y**ENMVTEIMSMGYER(E) |  | 4 | 13.40 |
| Vesicle-fusing ATPase | [Q9QUL6](http://www.uniprot.org/uniprot/Q9QUL6) | ([Ghesquiere et al., 2009](#_ENREF_7)) | (R)GDFLASLENDIkPAFGTNQEDYAS**Y**IMNGIIk(W) |  | 5 | 13.11 |
|  |  |  | (K)D**Y**QSGQHVMVR(T) |  | 3 | 14.14 |
|  |  |  | (R)LLD**Y**VPIGPR(F) |  | 2 | 12.31 |
| V-type proton ATPase subunit B, brain isoform |  |  | (K)AVVGEEALTSDDLL**Y**LEFLQk(F) |  | 6 | 15.65 |
|  |  |  | (R)I**Y**PEEMIQTGISAIDGMNSIAR(G) |  | 3 | 12.72 |

*Dimethylated lysines and carbamidomethylated cysteines are represented by under case letters, and nitration sites are represented by colored letters.

Aulak, K.S., Miyagi, M., Yan, L., West, K.A., Massillon, D., Crabb, J.W., and Stuehr, D.J. (2001). Proteomic method identifies proteins nitrated in vivo during inflammatory challenge. Proc Natl Acad Sci U S A *98*, 12056-12061.

Buchczyk, D.P., Grune, T., Sies, H., and Klotz, L.O. (2003). Modifications of glyceraldehyde-3-phosphate dehydrogenase induced by increasing concentrations of peroxynitrite: early recognition by 20S proteasome. Biol Chem *384*, 237-241.

Castegna, A., Thongboonkerd, V., Klein, J.B., Lynn, B., Markesbery, W.R., and Butterfield, D.A. (2003). Proteomic identification of nitrated proteins in Alzheimer's disease brain. J Neurochem *85*, 1394-1401.

Crow, J.P., Beckman, J.S., and McCord, J.M. (1995). Sensitivity of the essential zinc-thiolate moiety of yeast alcohol dehydrogenase to hypochlorite and peroxynitrite. Biochemistry *34*, 3544-3552.

Di Stasi, A.M., Mallozzi, C., Macchia, G., Petrucci, T.C., and Minetti, M. (1999). Peroxynitrite induces tryosine nitration and modulates tyrosine phosphorylation of synaptic proteins. J Neurochem *73*, 727-735.

Fan, X., Wang, J., Soman, K.V., Ansari, G.A., and Khan, M.F. (2011). Aniline-induced nitrosative stress in rat spleen: proteomic identification of nitrated proteins. Toxicol Appl Pharmacol *255*, 103-112.

Ghesquiere, B., Colaert, N., Helsens, K., Dejager, L., Vanhaute, C., Verleysen, K., Kas, K., Timmerman, E., Goethals, M., Libert, C.*, et al.* (2009). In vitro and in vivo protein-bound tyrosine nitration characterized by diagonal chromatography. Mol Cell Proteomics *8*, 2642-2652.

Ghosh, S., Janocha, A.J., Aronica, M.A., Swaidani, S., Comhair, S.A., Xu, W., Zheng, L., Kaveti, S., Kinter, M., Hazen, S.L.*, et al.* (2006). Nitrotyrosine proteome survey in asthma identifies oxidative mechanism of catalase inactivation. J Immunol *176*, 5587-5597.

Giasson, B.I., Duda, J.E., Murray, I.V., Chen, Q., Souza, J.M., Hurtig, H.I., Ischiropoulos, H., Trojanowski, J.Q., and Lee, V.M. (2000). Oxidative damage linked to neurodegeneration by selective alpha-synuclein nitration in synucleinopathy lesions. Science *290*, 985-989.

Gokulrangan, G., Zaidi, A., Michaelis, M.L., and Schoneich, C. (2007). Proteomic analysis of protein nitration in rat cerebellum: effect of biological aging. J Neurochem *100*, 1494-1504.

Kanski, J., Behring, A., Pelling, J., and Schoneich, C. (2005a). Proteomic identification of 3-nitrotyrosine-containing rat cardiac proteins: effects of biological aging. Am J Physiol Heart Circ Physiol *288*, H371-381.

Kanski, J., Hong, S.J., and Schoneich, C. (2005b). Proteomic analysis of protein nitration in aging skeletal muscle and identification of nitrotyrosine-containing sequences in vivo by nanoelectrospray ionization tandem mass spectrometry. J Biol Chem *280*, 24261-24266.

Koeck, T., Levison, B., Hazen, S.L., Crabb, J.W., Stuehr, D.J., and Aulak, K.S. (2004). Tyrosine nitration impairs mammalian aldolase A activity. Mol Cell Proteomics *3*, 548-557.

Lee, H.M., Reed, J., Greeley, G.H., Jr., and Englander, E.W. (2009). Impaired mitochondrial respiration and protein nitration in the rat hippocampus after acute inhalation of combustion smoke. Toxicol Appl Pharmacol *235*, 208-215.

Li, B., Held, J.M., Schilling, B., Danielson, S.R., and Gibson, B.W. (2011). Confident identification of 3-nitrotyrosine modifications in mass spectral data across multiple mass spectrometry platforms. J Proteomics *74*, 2510-2521.

Miyagi, M., Sakaguchi, H., Darrow, R.M., Yan, L., West, K.A., Aulak, K.S., Stuehr, D.J., Hollyfield, J.G., Organisciak, D.T., and Crabb, J.W. (2002). Evidence that light modulates protein nitration in rat retina. Mol Cell Proteomics *1*, 293-303.

Piroddi, M., Palmese, A., Pilolli, F., Amoresano, A., Pucci, P., Ronco, C., and Galli, F. (2011). Plasma nitroproteome of kidney disease patients. Amino Acids *40*, 653-667.

Reed, T.T., Owen, J., Pierce, W.M., Sebastian, A., Sullivan, P.G., and Butterfield, D.A. (2009a). Proteomic identification of nitrated brain proteins in traumatic brain-injured rats treated postinjury with gamma-glutamylcysteine ethyl ester: insights into the role of elevation of glutathione as a potential therapeutic strategy for traumatic brain injury. J Neurosci Res *87*, 408-417.

Reed, T.T., Pierce, W.M., Jr., Turner, D.M., Markesbery, W.R., and Butterfield, D.A. (2009b). Proteomic identification of nitrated brain proteins in early Alzheimer's disease inferior parietal lobule. J Cell Mol Med *13*, 2019-2029.

Sacksteder, C.A., Qian, W.J., Knyushko, T.V., Wang, H., Chin, M.H., Lacan, G., Melega, W.P., Camp, D.G., 2nd, Smith, R.D., Smith, D.J.*, et al.* (2006). Endogenously nitrated proteins in mouse brain: links to neurodegenerative disease. Biochemistry *45*, 8009-8022.

Smallwood, H.S., Galeva, N.A., Bartlett, R.K., Urbauer, R.J., Williams, T.D., Urbauer, J.L., and Squier, T.C. (2003). Selective nitration of Tyr99 in calmodulin as a marker of cellular conditions of oxidative stress. Chem Res Toxicol *16*, 95-102.

Stevens, S.M., Jr., Prokai-Tatrai, K., and Prokai, L. (2008). Factors that contribute to the misidentification of tyrosine nitration by shotgun proteomics. Mol Cell Proteomics *7*, 2442-2451.

Sultana, R., Poon, H.F., Cai, J., Pierce, W.M., Merchant, M., Klein, J.B., Markesbery, W.R., and Butterfield, D.A. (2006). Identification of nitrated proteins in Alzheimer's disease brain using a redox proteomics approach. Neurobiol Dis *22*, 76-87.

Sultana, R., Reed, T., Perluigi, M., Coccia, R., Pierce, W.M., and Butterfield, D.A. (2007). Proteomic identification of nitrated brain proteins in amnestic mild cognitive impairment: a regional study. J Cell Mol Med *11*, 839-851.

Suzuki, Y., Tanaka, M., Sohmiya, M., Ichinose, S., Omori, A., and Okamoto, K. (2005). Identification of nitrated proteins in the normal rat brain using a proteomics approach. Neurol Res *27*, 630-633.

Turko, I.V., Li, L., Aulak, K.S., Stuehr, D.J., Chang, J.Y., and Murad, F. (2003). Protein tyrosine nitration in the mitochondria from diabetic mouse heart. Implications to dysfunctional mitochondria in diabetes. J Biol Chem *278*, 33972-33977.

Tyther, R., Ahmeda, A., Johns, E., and Sheehan, D. (2007). Proteomic identification of tyrosine nitration targets in kidney of spontaneously hypertensive rats. Proteomics *7*, 4555-4564.

Uda, M., Kawasaki, H., Shigenaga, A., Baba, T., and Yamakura, F. (2012). Proteomic analysis of endogenous nitrotryptophan-containing proteins in rat hippocampus and cerebellum. Biosci Rep *32*, 521-530.

Vattemi, G., Mechref, Y., Marini, M., Tonin, P., Minuz, P., Grigoli, L., Guglielmi, V., Klouckova, I., Chiamulera, C., Meneguzzi, A.*, et al.* (2011). Increased protein nitration in mitochondrial diseases: evidence for vessel wall involvement. Mol Cell Proteomics *10*, M110 002964.

Vrljic, M., Strop, P., Hill, R.C., Hansen, K.C., Chu, S., and Brunger, A.T. (2011). Post-translational modifications and lipid binding profile of insect cell-expressed full-length mammalian synaptotagmin 1. Biochemistry *50*, 9998-10012.

Xiao, G.G., Nel, A.E., and Loo, J.A. (2005). Nitrotyrosine-modified proteins and oxidative stress induced by diesel exhaust particles. Electrophoresis *26*, 280-292.

Zhang, Q., Qian, W.J., Knyushko, T.V., Clauss, T.R., Purvine, S.O., Moore, R.J., Sacksteder, C.A., Chin, M.H., Smith, D.J., Camp, D.G., 2nd*, et al.* (2007). A method for selective enrichment and analysis of nitrotyrosine-containing peptides in complex proteome samples. J Proteome Res *6*, 2257-2268.
